# Cost-effectiveness of bubble continuous positive airway pressure in treating severe pneumonia and hypoxaemia in under-five children in Ethiopia

**DOI:** 10.1371/journal.pone.0352122

**Published:** 2026-06-23

**Authors:** Abdi Gari Negasa, Firew Tekle Bobo, Meseret Gebre, Tsinuel Nigatu, Mirkuzie Woldie, Mideksa Adugna Koricho, Mohammod J. Chisti, Peter Berman, Girmaye Dinsa

**Affiliations:** 1 School of Public Health, College of Health and Medical Science, Haramaya University, Harar, Ethiopia; 2 Centre of Disability Research and Policy (CDRP), Faculty of Medicine and Health, The University of Sydney, Sydney, Australia; 3 Armauer Hansen Research Institute (AHRI), Addis Ababa, Ethiopia; 4 Harvard T. H. Chan School of Public Health (HSPH), Boston, United States of America; 5 Institute of Health, Jimma University, Jimma, Ethiopia; 6 International Centre for Diarrheal Disease Research, Bangladesh (ICDDR, B), Dhaka, Bangladesh; Norbert Wiener University, PERU

## Abstract

**Background:**

Pneumonia is preventable and treatable, yet it remains the leading infectious cause of illness and death among under-five children. Bubble continuous positive airway pressure (bCPAP) offers a promising option for oxygen therapy combined with appropriate antibiotics and other supportive care. However, the cost-effectiveness of bCPAP in resource-limited settings such as Ethiopia is not documented. We aimed to evaluate the cost-effectiveness of bCPAP in treating severe pneumonia and hypoxaemia in under-five children in Ethiopia.

**Methods:**

We developed a decision-analytical model (decision tree) to determine the cost-effectiveness of a locally made bCPAP compared with the standard of care (WHO-recommended low-flow oxygen therapy) in general hospitals. Effectiveness was measured as the number of child deaths and disability adjusted life years (DALYs) averted. Cost data were extracted from published literature and local markets. The incremental cost-effectiveness ratio (ICER) was calculated and evaluated against the willingness-to-pay (WTP) thresholds set at multiples (0.34, 1, and 3) of Ethiopia’s GDP per capita. Sensitivity analyses were performed to test the robustness of the results.

**Results:**

For every 10,000 children with severe pneumonia and hypoxaemia, providing oxygen using locally made bCPAP will save an additional 31 children compared to the standard of care. A locally made bCPAP has an ICER of 139.5 USD per DALY averted. These results were robust in the sensitivity analysis performed, showing a 100% probability of being cost-effective at one times the GDP per capita of Ethiopia.

**Conclusion:**

A locally made bCPAP is a highly cost-effective intervention for treating severe pneumonia and hypoxaemia in under-five children in Ethiopian general hospitals. These findings provide critical evidence for decision-makers to support and scale-up use of bCPAP in Ethiopia and other similar low and middle income countries.

## Introduction

In 2019, pneumonia killed 740,180 children before they reached their fifth birthday. It is a significant global health concern and remains a leading infectious cause of morbidity and mortality, accounting for 14% of all deaths among children under-five years of age [1].

Severe pneumonia in children is defined by clinical signs of pneumonia (such as cough or difficulty breathing) combined with danger signs or hypoxaemia. Specifically, it includes at least one of: central cyanosis, SpO_2_ < 90%, severe respiratory distress (e.g., grunting, very severe chest indrawing), or general danger signs like inability to breastfeed/drink, lethargy/unconsciousness, or convulsions [2]. The severity of pneumonia is often exacerbated by hypoxaemia, a condition characterized by low blood oxygen levels (less than 90% at room air measured by pulse oximeter), presence of which significantly increases the risk of fatal outcomes compared to those without hypoxaemia [3–5].

While pneumonia affects children and families worldwide, the highest death rates are concentrated in southern Asia and sub-Saharan Africa [1,6]. In Ethiopia, pneumonia is among the major reasons of hospital visit and mortality in under 5 children [7]. The pooled magnitude of pneumonia among under-five children was in Ethiopia was 18.03% [8] and mortality rate was relatively high with 16.09% in severe pneumonia [9].

The majority of pneumonia-related deaths in children are preventable or treatable with simple and inexpensive interventions [10]. Effective strategies include prevention through basic public health measures, such as vaccination and improved nutrition, and treatment with low-cost medications and supportive care. However, the management of severe pneumonia and hypoxaemia, especially in resource-limited settings, poses a significant challenge [3].

The WHO recommends low flow oxygen therapy, antibiotics, and supportive care for treating severe pneumonia and hypoxaemia [10]. In developed countries, low flow oxygen therapy or continuous positive airway pressure (CPAP) is widely used in intensive care units (ICUs) for children with moderate to severe respiratory distress [11]. The most common method to deliver CPAP is through a mechanical ventilator, which is expensive and unavailable in most health facilities in Ethiopia. Another technique involves using a humidified high-flow mixture of air and oxygen delivered via a nasal cannula, which is also expensive and inaccessible [3,11].

Bubble CPAP (bCPAP) offers promising alternative oxygen therapy for treating severe paediatric pneumonia, especially in resource-limited settings. This low-cost intervention can be implemented using simple materials and has been clinically effective in many low-income countries [12,13]. Studies in Bangladesh and Ethiopia have shown that children receiving bCPAP have significantly better outcomes compared to those receiving standard care [3,4]. However, findings from Ghana and Malawi did not demonstrate the same effectiveness [13,14]. In Ghana, bCPAP showed no difference in outcomes compared to standard care among children under-five, although a post-hoc analysis showed a survival benefit of bCPAP among infants. In Malawi, the lack of daily physician (paediatrician) supervision was identified as a key factor influencing the results.

While a locally made bCPAP has demonstrated its effectiveness in Ethiopia, its cost-effectiveness remains to be fully evaluated [4,15]. Currently, the Ethiopian Ministry of Health (MoH) has not yet fully incorporated bCPAP into national pediatric clinical guidelines. Understanding the economic viability of bCPAP is essential for its sustainable integration into healthcare systems and its potential to improve outcomes and reduce overall healthcare costs. Cost-effectiveness evidence is essential for guiding decisions on adopting and scaling up bCPAP, while also strengthening its case for recognition and large-scale implementation. To that end, we evaluated the cost-effectiveness of bCPAP in general hospital settings in Ethiopia.

## Materials and methods

### Model design

**Fig 1:**
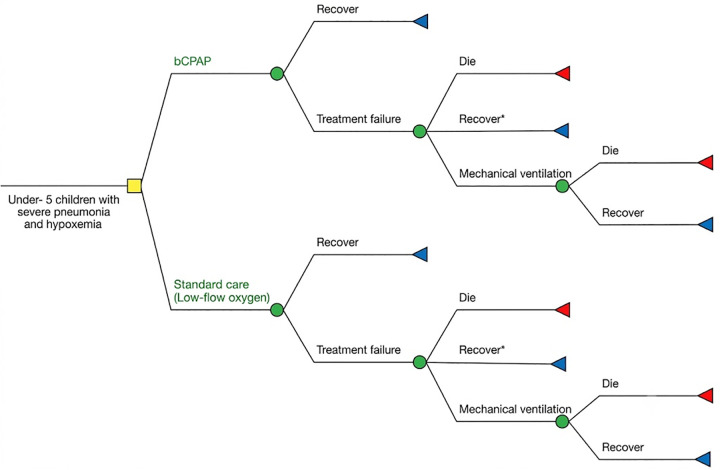
Decision tree model structure. bCPAP = bubble Continuous Positive Airway Pressure; Square box is the decision node; Circles are chance nodes, and the triangles show outcomes. Recover*= recovery from treatment failure after escalated oxygen therapy.

This cost-effectiveness analysis was conducted based on a prior, pragmatic cluster-randomized controlled trial on the use of locally made bCPAP compared with WHO-recommended low-flow oxygen therapy in treating children with severe pneumonia and hypoxaemia in 12 general (secondary) hospitals in Ethiopia [4]. This cost-effectiveness analysis was conducted in December 2024, the trial ran from June 8, 2021, to July 27, 2022. We used a decision analytical model (decision tree) to compare the cost-effectiveness of low-flow and bCPAP oxygen therapy for a hypothetical cohort of children aged 1–59 months (Fig 1). We defined the time horizon from the diagnosis and admission of severe pneumonia with hypoxaemia to either hospital discharge or death. The long-term costs and benefit of the intervention were not included in the study. This study is reported in accordance with the Consolidated Health Economic Evaluation Reporting Standards (CHEERS 2022) statement (S1 Table in S1 File) [16].

### Description of interventions

The treatment of severe paediatric pneumonia requires prompt medical attention from a healthcare professional. The first-line treatment typically includes parenteral ampicillin (or penicillin) combined with gentamicin. The WHO Classification and Treatment of Pneumonia in Children emphasize timely intervention and supportive care, including oxygen therapy and proper fluid management, to improve outcomes for affected children [10]. Hypoxaemia caused by pneumonia is a significant risk factor for death, increasing the risk up to five times compared to children without hypoxaemia. Therefore, children with severe pneumonia complicated by hypoxaemia require immediate treatment with oxygen therapy. This study compares two methods of providing oxygen therapy: low-flow and bCPAP oxygen therapy, as described below.

- **Standard of care** (**Low-flow oxygen therapy**): Low-flow oxygen therapy via nasal prongs is the primary method of oxygen delivery in all hospitals in Ethiopia. Standard of care involves starting oxygen flow set at 1 Littre (L)/minute and increasing every 5 minutes up to a maximum of 2 L/minute for children aged 1 month to 2 years and 4 L/minute for children older than 2 years to achieve SpO2 of 90% or more.
- **Intervention (bCPAP oxygen therapy**): bCPAP was assembled on site using standard nasal oxygen prongs, plastic tubing customarily used for administration of intravenous fluids (Opso Saline, Dhaka, Bangladesh), and a transparent plastic bottle filled with water to the 10 cm mark. Inspiratory gas flow was provided by oxygen concentrators (230 V, 50 Hz, 3·2 A; DeVilbiss Healthcare, Port Washington, NY, USA). bCPAP was initiated at a depth 5 cm below the water level inside the plastic bottle. The expiratory tubing was inserted with 5 L/min of oxygen delivered from the oxygen source. If peripheral oxygen saturation (SpO2) of 90% or more was not achieved within 5 min, both the depth of the expiratory tubing and oxygen flow were titrated every 5 min to achieve SpO2 of 90% or more [4].

The antibiotic treatment, feeding, and routine assessment of vital signs were conducted as directed by the Ethiopian National Severe Pneumonia Treatment Guidelines at the general hospital level [17]. Those patients who treated as outpatient, started treatment in other hospitals, children who had any one of the following: congenital heart disease, asthma, upper airway obstruction, tracheostomy, pneumothorax, or requirement for mechanical ventilation at admission as decided by the clinician were excluded from the study. In both group reassessment was done at 1 hour and 4 hours after initiation, and then, for patients who respond, every 4 hours during the first 24 hours, followed by every 8 hours after that [4]. Treatment failure in both groups was defined as the presence of severe hypoxaemia (SpO2 < 85%) at any time after at least 1 hour of intervention plus signs of respiratory distress, or development of an indication for mechanical ventilation, or death during the hospital stay, or leaving the hospital against medical advice while still on study intervention. Indication for mechanical ventilation included clinical worsening while on bCPAP or low-flow oxygen therapy (after at least 1 h); respiratory failure (apnoea or respiratory arrest); inadequate ventilation or inadequate oxygenation; cardiac insufficiency or shock; or neurological dysfunction (central hypoventilation or frequent apnoea, Glasgow Coma Scale <8, and inability to protect airway) [4]. Children who experienced treatment failure were initially placed on escalated oxygen therapy, receiving high-flow oxygen through a face mask at a rate of 5–10 L/minute. Recovery after treatment failure was assumed because the trial reported that many patients showed improvement with escalated oxygen therapy [4]. Mechanical ventilation was considered only for those patients who did not respond to this escalated treatment. Although leaving the hospital against medical advice while still on study intervention was one of the composite measures of treatment failure in the trial, the clinical endpoint for those who left against medical advice is unknown, so we have not included it in this study.

### Model parameters and assumptions

#### Effectiveness of intervention.

Data regarding the effectiveness of bCPAP and low-flow oxygen were sourced from a pragmatic cluster-randomized controlled trial in Ethiopia [4] and similar studies, as summarized in Table 1. The trial compared locally made bCPAP with WHO-recommended low-flow oxygen therapy in children with severe pneumonia and hypoxaemia. The primary outcome of the trial was treatment failure. We evaluated the effectiveness of the intervention and the comparator in terms of treatment recovery rate and death rate as reported in the trial [4]. We converted deaths into DALYs using the average age of paediatric patients who died from severe pneumonia (11 months, according to the trial) and the life expectancy in Ethiopia at 1 year of age. We then discounted DALYs by 3% [18]. We used the recovery rate among patients put on mechanical ventilation from a study conducted in another country with a similar context [19].

**Table 1:** Effectiveness parameters.

Parameters	Base value	Range for sensitivity analysis	Distribution type assumed	Reference
Probability of treatment failure among bCPAP group	0.008	0.004 to 0.06	Beta (5, 615)	[3,4]
Probability of treatment failure among Low-flow oxygen therapy group	0.034	0.017 to 0.24	Beta (21, 599)	[3,4]
Probability of death among treatment failures in bCPAP group	0.2	0.1 to 0.3	Beta (1 4)	[3,4]
Probability of death among treatment failures in Low-flow oxygen therapy group	0.29	0.14 to 0.43	Beta (6 15)	[3,4]
Probability of being on Mechanical ventilation in bCPAP group	0.06	0.03 to 0.09	Beta (3, 76)	[3]
Probability of being on Mechanical ventilation in Low-flow oxygen therapy group	0.16	0.08 to 0.24	Beta (11, 56)	[3]
Probability of Recovery after mechanical ventilation	0.447	0.3 to 0.63	Beta (53, 58)	[19,20]
Average Duration of intervention in Low-flow oxygen therapy group	32hrs	24–48	Normal	[4]
Average Duration of intervention in bCPAP group	24hrs	20–32	Normal	[4]
Average time of hospital stay in Low-flow oxygen therapy group	62.2hrs	44·5 to 96·0	Normal	[4]
Average time of hospital stay in bCPAP group	53hrs	44·9 to 76·0	Normal	[4]

*bCPAP = bubble Continuous Positive Airway Pressure. hrs = hours. For beta distribution (α) alpha and (β) beta value was specified in the bracket, respectively.*

### Cost input parameters

We estimated each unit cost input parameter based on the best available sources. To identify the types of resource inputs, we reviewed the severe pneumonia management guidelines for general hospitals in Ethiopia [17], published articles, and we asked experts who have experienced in locally preparing bCPAP. We adopted a restricted societal perspective. The ‘restricted societal’ perspective includes all direct medical costs regardless of who funds it, but not productivity loss or other expenses. Accordingly, all direct medical costs, including the cost of hospitalization, staff time, locally producing bCPAP, oxygen supply, and mechanical ventilation were included in the analysis.

We used previously published literature in Ethiopia to determine the cost of hospitalization for severe pneumonia, factoring in expenses such as transportation, registration, laboratory tests, medications, supplies, and hospital bed fees. We then adjusted these costs to the 2022 US dollar equivalent using the consumer price index of Ethiopia [21].

To estimate the unit cost of health workers’ time spent diagnosing and treating severe pneumonia and hypoxaemia, we used a health worker-to-patient ratio based on the trial protocol: one nurse per six patients, one general practitioner per 18 patients, and one paediatrician per hospital [4]. We calculated health workers’ daily salary, including off-duty hour payments. We divided the product by health worker- to-patient ratio for each health worker category to get personnel time per patient per day. Finally, personnel time per patient per day was multiplied by the average time of hospital admission days.

We estimated the cost of locally preparing bCPAP based on the local market price, considering the cost of nasal oxygen prongs, plastic tubes, and a transparent plastic bottle from a local market and expert in locally preparing bCPAP. We used published literature from countries with similar contexts to determine the cost of oxygen supply. According to the study from Gambia, the average cost of oxygen from a concentrator source was 1.0332 USD per 1,000 liters (adjusted to 2022 USD) [22]. We then multiplied this cost by the oxygen flow rate (based on each group’s L/min of oxygen) and the average duration of each intervention to obtain the unit cost of oxygen supply per episode (Appendix in S2 File). We used the cost of mechanical ventilation for COVID-19 patients in Ethiopia as a proxy cost estimate for the cost of mechanical ventilation in treatment failure for severe pneumonia [23]. All costs were adjusted to 2022 USD using the consumer price index [21]. In Table 2-unit cost parameters are listed.

**Table 2:** Unit cost parameters.

Unit cost Parameters	Base value (US $)	Range for sensitivity analysis	Distribution type assumed	Reference
Total cost of hospitalization (per episode)	101.4	50.7 to 152.1	Gamma (1.38, 46.17)	[24]
Transportation	12.5	–	–	[24]
Registration/consultation	4.3	–	–	[24]
Laboratory	14.68	–	–	[24]
Medicines and supplies	57.06	–	–	[24]
Hospital bed	25.38	–	–	[24]
Staff time cost (per-patient per episode)	2.6	1.3 to 3.9	Point estimate	Estimated
Cost of locally preparing bCPAP*	2	1–3	Point estimate	Estimated
Cost of oxygen supply (per 1000 liter) **	1.0332	0.5166 to 1.5498	Gamma (1, 1.0332)	[25,26]
Cost of mechanical ventilation (per patient per day)	158	79–237	Gamma (1, 158)	[23]

NB. * Only for bCPAP group. ** Cost of oxygen supply per liter was multiplied by the mean duration of intervention in each group and according to flow rate for both Low-flow oxygen therapy and bCPAP. For gamma distribution, (α) alpha and (β) beta value was specified in the bracket, respectively.

### Data analysis

#### Cost-effectiveness analysis.

We calculated ICER between Low-flow oxygen therapy and bCPAP intervention. This study defined ICER as (Cost _bCPAP_- Cost _Low-flow oxygen_)/ (Effect _bCPAP_ - Effect _Low-flow oxygen_). The cost per child death averted and cost per DALYs averted were reported. The intervention was deemed cost-effective if the cost per DALY averted is less than three times GDP per capita and very cost-effective if it is less than one times GDP per capita according to the WHO-CHOICE criteria [18]. However, this threshold was criticized as it set such a low bar for cost-effectiveness that very few interventions with evidence of efficacy can be ruled out [27]. To ensure a more conservative approach, we adopted thresholds based on health expenditure per capita and the Human Development Index (HDI), as suggested by Daroudi et al. [28]. Given that Ethiopia is classified as a low HDI country by the United Nations Development Programme (UNDP) [21], we used the cost per DALY averted threshold set at 0.34 times the GDP per capita suggested for low HDI countries [28]. The GDP per capita of Ethiopia in 2022 was $ 856 [15].

#### Sensitivity analysis.

We implemented both deterministic and probabilistic sensitivity analysis (PSA) to check the robustness of the results. A series of one-way sensitivity analyses were conducted by varying key input parameters once at a time over high value and low value to determine the effect of each parameter on estimated ICER and presented in a tornado diagram. To test the joint effect of these parameter uncertainties, a PSA was conducted by using a Monte Carlo simulation with 10,000 iterations. Cost parameter values were specified by gamma distribution (range of ± 50%) and beta distribution for probabilities (a range taken from published literature; if unavailable, we varied by ± 50%, constrained between 0 and 1) (Tables 1 and 2). PSA was presented in a cost-effectiveness acceptability curve. Data analysis was performed using Microsoft Excel, with Visual Basic for Applications (VBA) macros used for sensitivity analysis.

### Ethics statement

This study is based on the analysis of previously conducted studies and publicly available, aggregated secondary data. It does not contain any new studies with human participants or animals performed by any of the authors. Original studies from which the data were derived were conducted in accordance with ethical standards, including obtaining informed consent from participants. The authors did not have access to information that could identify individual participants during or after data collection. Data were collected anonymously, and no identifying markers were recorded. Because this study utilized anonymized, publicly available data, the requirement for direct informed consent was waived, and no further institutional review board approval was required for this secondary analysis.

## Results

### Base case analysis

For every 10,000 children with severe pneumonia and hypoxaemia, the provision of oxygen using locally made bCPAP will save an additional 31 children compared to standard of care. As shown in Table 3, compared to the standard of care, a locally made bCPAP has an ICER of 139.5 US$ per DALYs averted. This intervention is highly cost-effective when considering one times the GDP per capita of Ethiopia in 2022 ($856) based on the WHO-CHOICE threshold. This intervention remains cost-effective even when the highly conservative 0.34 times GDP per capita ($291) is used.

**Table 3:** Costs, Deaths averted per 10,000, DALYs, and ICER of Low-flow oxygen therapy and bCPAP intervention.

Interventions	Costs (US$)	Inc. Cost	Deaths per 10,000	Deaths averted per 10,000	DALYs	Inc. DALYs averted	ICER(Cost per DALYs)
Low-flow oxygen	110.83	Ref.	34	Ref.	0.1163	Ref.	Ref.
bCPAP	125.33	14.50	3	31	0.0124	0.1039	139.49

*Costs and DALYs were presented per patient. Inc.: Incremental. Ref.: Reference intervention, bCPAP: bubble Continuous Positive Airway Pressure, DALYs: Disability-Adjusted Life Years, ICER: Incremental Cost-Effectiveness Ratio. Inc. DALYs are documented as positive values, but the calculation gives a negative value because smaller values of DALYs are the preferred outcome.*

### Sensitivity analysis

**Fig 2:**
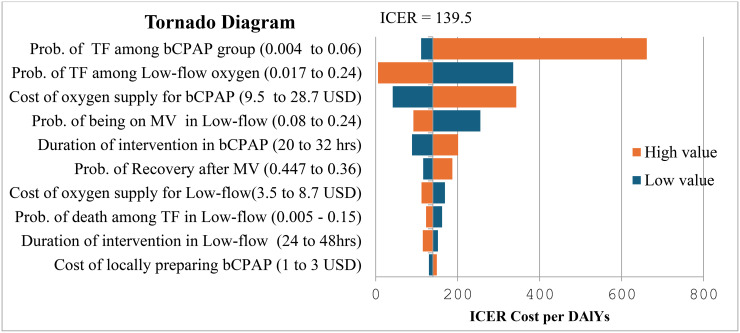
A Tornado diagram of bCPAP compared to Low-flow oxygen therapy. Prob. Probability, bCPAP: bubble Continuous Positive Airway Pressure, ICER: Incremental Cost-Effectiveness Ratio, TF: Treatment Failure, MV: Mechanical Ventilation.

The results of one-way sensitivity analysis were presented in a Tornado diagram in Fig 2. The most influential parameters were the probability of treatment failure in the bCPAP group and Low-flow oxygen therapy group, the probability of being on mechanical ventilation in the Low-flow oxygen therapy group, the cost of oxygen supply in the bCPAP group, the duration of bCPAP intervention, and the cost of oxygen supply in the bCPAP group. Although changes in these parameters affected the base case, the ICER remained within a range of less than one times the GDP per capita of Ethiopia.

**Fig 3:**
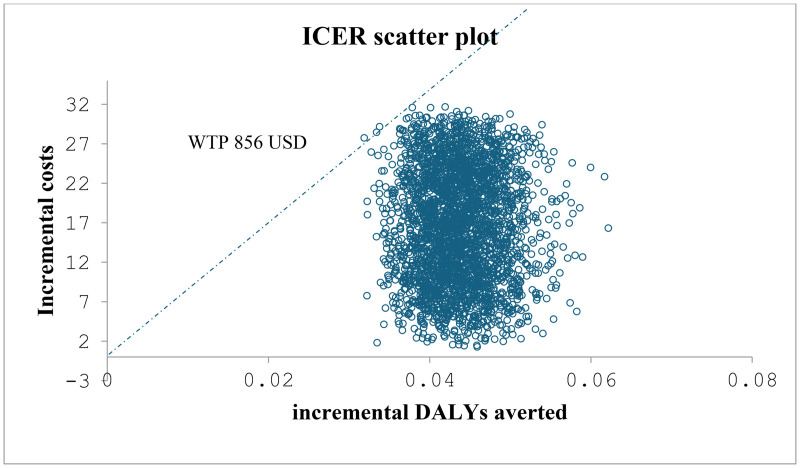
ICER scatter plot for bCPAP compared to low-flow oxygen therapy. The dashed line indicates WTP at one times ($856) GDP per capita. The dots indicates incremental costs and incremental DALYs averted by bCPAP compared to low-flow oxygen therapy based on Monte Carlo simulation (n=10,000).

**Fig 4:**
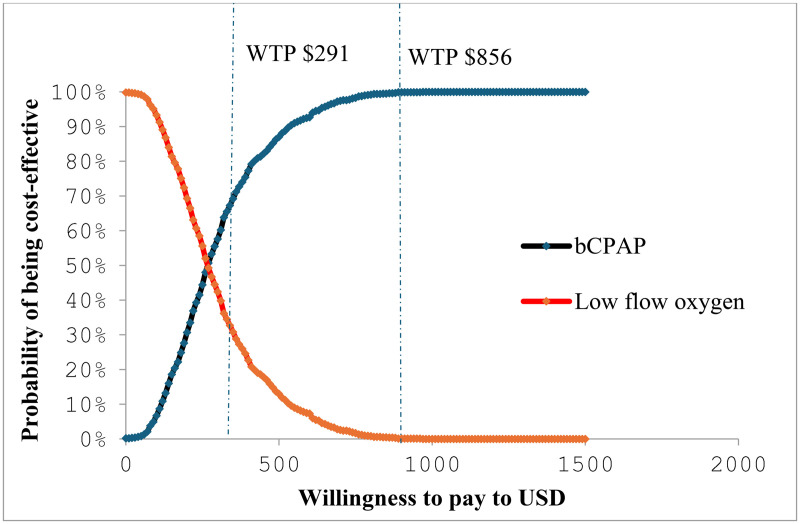
Cost-effectiveness acceptability curve for bCPAP compared to low-flow oxygen therapy. The dashed line indicates WTP at one times ($856) and 0.34 times ($291) GDP per capita of Ethiopia.

To assess the joint effect of all these key input parameters on the base case ICER, PSA was conducted using Monte Carlo simulation with 10,000 iterations (Figs 3 and 4). As shown in the ICER scatter plot and cost-effectiveness acceptability curve, bCPAP is 100% likely to be cost-effective at one times the GDP per capita of Ethiopia ($856) when compared to WHO-recommended low-flow oxygen therapy.

## Discussion

This study aimed to assess the cost-effectiveness of the locally made bCPAP in the Ethiopian general hospital. Following a pragmatic cluster-randomised controlled trial that proved the device’s effectiveness [4], robust economic evidence is now essential to guide national adoption and policy decisions. This study found that bCPAP is highly cost-effective compared to standard care (low-flow oxygen therapy) for treating severe pneumonia and hypoxaemia in children under five in Ethiopia. Our results suggest that bCPAP oxygen therapy provides substantial clinical benefits and is economically feasible, particularly in low-resource settings such as Ethiopia, where childhood pneumonia continues to be a major cause of mortality.

When compared against the WHO-CHOICE threshold, which is equivalent to one times the GDP per capita of a low-income country (estimated at $856 for Ethiopia in 2022), bCPAP’s cost-effectiveness is well within the acceptable range, suggesting that the investment in bCPAP oxygen therapy is a justifiable use of limited health resources. Even considering the more conservative threshold of $291 per DALY averted, representing 0.34 times GDP per capita for low HDI countries, bCPAP oxygen therapy still falls comfortably within this range [29].

The cost-effectiveness of bCPAP oxygen therapy observed in this study is comparable to a range of other critical child health interventions in resource-limited settings, such as the vaccination programs, which have been shown to range from $7 to $438 per DALYs averted, depending on the specific vaccine and setting [30].

While our study supports the clinical efficacy of bCPAP oxygen therapy and establishes that it is a highly cost-effective intervention in Ethiopia, it is important to contextualize these findings within the broader evidence base, particularly in LMICs. Previous studies in various LMIC settings have produced mixed results regarding the clinical effectiveness of bCPAP, indicating the need for a cautious interpretation of its potential impact [3,4,13,14,31].

Studies from Bangladesh and Ethiopia strongly support bCPAP as an effective intervention [3,4]. In Bangladesh, a trial conducted in an intensive care unit (ICU) where children received daily physician supervision and care from trained nurses demonstrated that bCPAP oxygen therapy significantly reduced mortality compared to standard low-flow oxygen therapy [3]. Similarly, recent findings from Ethiopia [4], on which our study is based, showed high efficacy of bCPAP in reducing mortality and adverse outcomes. The bCPAP intervention in these studies was overseen by paediatricians and trained healthcare workers, highlighting its potential in settings with sufficient clinical oversight and well-trained staff.

However, contrasting evidence from trials conducted in Malawi and Ghana raises questions [13,14]. In Malawi, a trial conducted in a setting where trained nurses provided care, with only telephone consultations from paediatricians, found no significant difference in outcomes between children treated with bCPAP and those receiving low-flow oxygen [14]. The study from Ghana, an RCT conducted under daily physician oversight, found lower mortality benefit among infants (<12 months of age) with undifferentiated respiratory distress receiving bCPAP compared to low flow oxygen therapy, although there was no difference in primary mortality outcomes among under-five children between the two interventions [13]. These findings suggest that the effectiveness of bCPAP depends on the healthcare delivery context and the level of clinical supervision.

A systematic review and meta-analysis published in 2021 concluded that the efficacy of bCPAP is context-dependent [31]. The review found limited evidence to support bCPAP’s effectiveness in reducing mortality and adverse events, and the certainty of this evidence was deemed low. While bCPAP may offer benefits in certain settings, these findings suggest that its overall effectiveness in LMICs remains uncertain and could vary depending on factors such as healthcare infrastructure, availability of trained personnel, and access to other essential resources like oxygen and monitoring equipment.

Our findings align with prior cost-effectiveness studies conducted in Malawi [32]. In 2017, Kortz et al. demonstrated that bCPAP was a cost-effective intervention for treating severe pneumonia and hypoxaemia in under-five children. Another study from Malawi established that bCPAP was also cost-effective in providing ventilatory support to neonates, further validating its economic viability in resource-limited settings [33].

Interestingly, while earlier studies supported the cost-effectiveness of bCPAP, subsequent clinical evidence from Malawi provides less supportive evidence of its efficacy [14,32]. One possible explanation for the discrepancy between the cost-effectiveness studies and the clinical trial outcomes could be related to the source of the effectiveness data used in the cost-effectiveness analysis. In the earlier Malawian study, the effectiveness data were derived from research conducted in Bangladesh, where bCPAP was highly effective in an intensive care unit setting with close physician supervision and trained nurses [32].

This study has limitations that should be considered when interpreting the results. First, we relied on secondary cost data, which may have led to inaccuracies, such as double counting or omitting certain costs. The use of secondary data inherently carries the risk of variability in cost estimates, particularly when different studies use varying methodologies, healthcare resource utilization rates, or cost components. For example, our analysis might not have captured all the indirect costs associated with bCPAP oxygen therapy, such as maintenance, training, or the potential impact of equipment failure.

However, we have made significant efforts to mitigate these limitations by using secondary cost data from published studies related to the Ethiopian context and considering a wide sensitivity range (up to ±50% of the base value) in our analysis. We prioritized data from studies with robust methodologies and transparent cost reporting to ensure our cost-effectiveness estimates are as accurate and relevant as possible.

Second, severe paediatric pneumonia often has long-term health impacts that were not accounted for in our analysis. Due to the lack of long-term data, we could not consider the potential long-term costs and benefits associated with bCPAP intervention, such as the reduction in long-term morbidity, improved quality of life, or future healthcare costs saved.

With its favorable cost-effectiveness profile, bCPAP oxygen therapy offers a scalable solution that can be integrated into existing healthcare services for paediatric pneumonia management. As Ethiopia continues to face challenges in providing access to effective advanced respiratory support interventions, which is often expensive, bCPAP oxygen therapy presents an affordable alternative that can be deployed in district and regional hospitals with limited resources. However, given the mixed evidence on its effectiveness across different settings, additional evidence is needed before large-scale implementation can be considered.

Scaling up of bCPAP oxygen therapy in Ethiopia and similar settings would require several considerations. Context-specific implementation studies, especially those assessing bCPAP’s performance in rural and low-resource settings, are needed to ascertain the potential of this intervention to improve treatment outcomes at an affordable cost [31]. Furthermore, investments in healthcare worker training, ensuring the availability of essential equipment, and maintaining consistent oxygen supplies will be crucial to maximize the impact of this intervention.

Additional research is warranted to address the limitations identified in this study. Specifically, data analysis using a net regression approach and incorporating primary data on patient-specific costs and effects is essential to account for individual-level variation. Furthermore, future research should consider the long-term costs and impacts of bCPAP to assess its value over extended periods comprehensively.

## Conclusions

This study demonstrated that a locally made bCPAP is a highly cost-effective intervention for treating severe pneumonia and hypoxaemia in children under five in Ethiopia. The bCPAP’s favorable cost-effectiveness profile, along with its robustness to various input parameters, makes it a scalable solution that can be seamlessly integrated into existing healthcare services having monitored by nurses under the supervision of qualified physicians for pediatric pneumonia management in general hospitals in Ethiopia. These findings provide critical evidence for decision‑makers to support the scaled‑up rollout of oxygen‑related interventions, but this expansion must be explicitly linked to adequate training and ongoing supervision of health workers, reliable oxygen supply and equipment management, and systematic implementation monitoring to ensure that scale‑up improves access, safety, and clinical outcomes rather than creating undersupplied or poorly implemented services.

## Supporting information

S1 File Consolidated Health Economic Evaluation Reporting Standards 2022 (CHEERS 2022) Statement checklist. (DOCX)

S2 File Appendix. (DOCX)
