# Supplementary material for: Cost-effectiveness of bubble continuous positive airway pressure in treating severe pneumonia and hypoxaemia in under-five children in Ethiopia
Source: PLoS One. 2026 Jun 23;21(6):e0352122. doi: 10.1371/journal.pone.0352122 (PMC13289904; doi:10.1371/journal.pone.0352122)
Supplement: S2 File — (DOCX) [file pone.0352122.s002.docx]

**Supplementary File**

Figure: Decision tree model equation with parameters fitted with input parameters for base case scenario. A decision-analytic model was developed to compare the cost-effectiveness of bCPAP vs. low-flow oxygen. The model captures the clinical pathway from initial treatment to recovery, failure, or escalation to mechanical ventilation. Outcomes were modelled with health consequences expressed in Disability-Adjusted Life Years (DALYs) and costs adjusted to 2022 USD

**
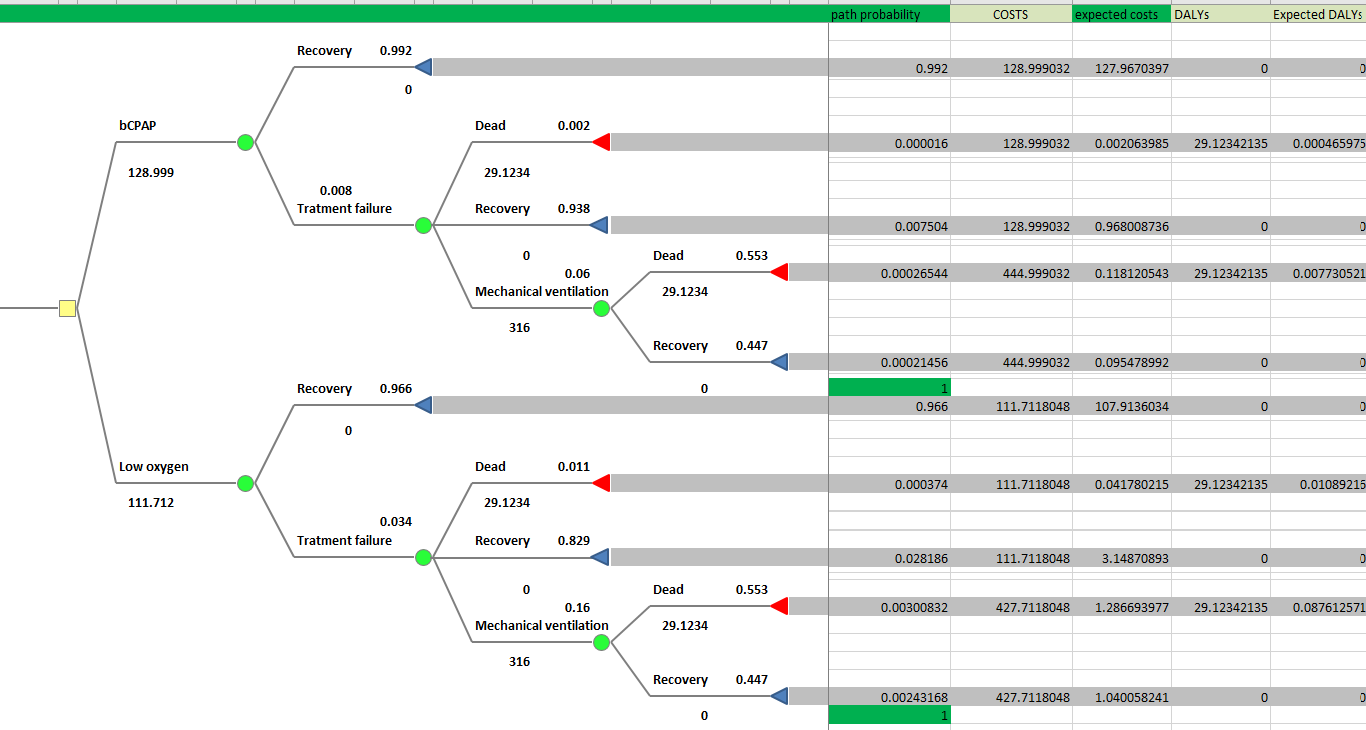
**

Figure 1: Decision tree model structure. *Note: bCPAP= bubble Continuous Positive Airway Pressure; Square box is the decision node; Circles are chance nodes, and the triangles show outcomes. Recover*= recovery from treatment failure after escalated oxygen therapy.*

**Oxygen cost calculation for bCPAP**

**The cost of oxygen cost was calculated as the following**

The average cost of oxygen from a concentrator source was 1.0332 USD per 1,000 liters (adjusted to 2022 USD). The average Duration of intervention in bCPAP group was 24hrs, the average oxygen flow rate for bCPAP group 5 L/min of oxygen. To calculate the unit cost of oxygen supply per episode for the bCPAP group, we follow these steps:

**1. Variables**

- Oxygen Cost: $1.0332 USD per 1,000 liters
- Flow Rate: 5 L/min
- Average Duration: 24 hours

**2. Calculate Total Volume of Oxygen Consumed**

First, we convert the duration into minutes and multiply by the flow rate:

Total Duration (minutes)=24 hours×60 minutes/hour=1,440 minutes

Total Liters=5 L/min×1,440 minutes=7,200 liters

**3. Calculate the Unit Cost per Episode**

Next, we calculate the cost based on the price per 1,000 liters:

Cost per liter= 1,000 liters/$1.0332=$0.0010332 per liter

Unit Cost=7,200 liters×$0.0010332 per liter

Unit Cost≈$7.439

Final Result: The unit cost of oxygen supply per episode for the bCPAP group is approximately $7.44 USD (adjusted to 2022 USD)

**Oxygen cost calculation for low flow oxygen**

The average cost of oxygen from a concentrator source was 1.0332 USD per 1,000 liters (adjusted to 2022 USD). The average Duration of intervention in bCPAP group was 62.2hrs, the average oxygen flow rate for low flow oxygen group 1 L/min of oxygen. To calculate the unit cost of oxygen supply per episode for the low flow oxygen group.

**1. Variables for Low-Flow Group**

- **Oxygen Cost:** 1.0332$ USD per 1,000 liters
- **Flow Rate:** 1L/min
- **Average Duration:** 62.2$ hours

**2. Step-by-Step Calculation**

**A. Total Duration in Minutes**

First, convert the average duration from hours to minutes:

62.2hours* 60 = 3,732 minutes

**B. Total Volume of Oxygen Consumed**

Multiply the flow rate by the total duration in minutes:

1 L/min * 3,732 minutes = 3,732 minutes

**C. Unit Cost per Episode**

Calculate the cost based on the price per 1,000$ liters:

Cost per liter = 1.0332/1,000 = 0.0010332 USD/liter . Unit Cost = 3,732liters * 0.0010332 **= 3.8559 USD**
