# Supplementary material for: Cost-effectiveness of bubble continuous positive airway pressure in treating severe pneumonia and hypoxaemia in under-five children in Ethiopia
Source: PLoS One. 2026 Jun 23;21(6):e0352122. doi: 10.1371/journal.pone.0352122 (PMC13289904; doi:10.1371/journal.pone.0352122)
Supplement: Attachment — Submitted filename: REVEWING ATTACHEMENTS.docx [file pone.0352122.s003.docx]

Dear Editor,

Thank you for inviting me to review "Cost-effectiveness of locally made bCPAP for severe pneumonia in Ethiopian children." I have completed my review and provide my detailed comments below.

**Overall Recommendation**

**MINOR REVISION REQUIRED**

**Summary**

The study addresses a critical public health question using robust RCT data. Methods are conceptually sound but lack transparency in parameter sourcing, model assumptions, and long-term impacts. With clarifications, this could become a strong candidate for publication.

Here is my comments:

1. **Abstract lacks precision on key metrics.** Current results state "31 children saved" but Table 1 shows 31 deaths averted (34 vs 3), which is accurate but needs clearer phrasing.
2. **Table numbering chaos.** Abstract/Results reference Table 1 for ICER, but effectiveness parameters are Table 1, costs Table 2.
3. **Typos and inconsistencies.** "hypoxeamia" → "hypoxaemia"; "Littre" → "litre"; "DAlYs" → "DALYs"
4. **Method:** the severe pneumonia definition is NOT clearly stated in the manuscript.
5. Transport $12.5 listed but not modeled. Clarify family costs
6. **Discussion:** Major Cuts Needed; remove repetitive trial summaries

I am happy to re-review the revised version and have no conflicts of interest.

Thank you for the opportunity to review this important work.

Best regards,

Emna Ben Jemia

Hospital-University Assistant in Pulmonology

Charles Nicolle Hospital, Tunis
