# Supplementary material for: Cost-effectiveness of bubble continuous positive airway pressure in treating severe pneumonia and hypoxaemia in under-five children in Ethiopia
Source: PLoS One. 2026 Jun 23;21(6):e0352122. doi: 10.1371/journal.pone.0352122 (PMC13289904; doi:10.1371/journal.pone.0352122)
Supplement: Attachment — Submitted filename: Point by point response to editor and reviewers.docx [file pone.0352122.s006.docx]

**Point by point response to academic editor and reviewers comment**

We would like to express our sincere gratitude to the editor and reviewers for their time and insightful feedback on this manuscript. Their thoughtful comments and constructive suggestions have significantly improved this work. We carefully reviewed each comment and have diligently modified our manuscript accordingly. In this response, we provide detailed justifications for the changes made, demonstrating how we have addressed the reviewers' concerns and incorporated their valuable suggestions. We deeply appreciate their dedication to enhancing the quality of this research.

We provided point by point response for each reviewer following the comments/questions in ***italics* f**ont type.

**Comments from editor**

1. The study would benefit from **greater conceptual and methodological clarity**. It is essential to define **severe pneumonia** precisely according to recognized criteria, ideally based on WHO standards, including danger signs and oxygen saturation below 90%. The **study period**, the type and recency of the published sources used, and the analytical model applied should also be described more clearly. In addition, the manuscript should provide a stronger justification for the comparability of the two interventions, better describe the clinical trial or source evidence used, and explain more explicitly the assumptions underlying the cost-effectiveness analysis.

***Response***

- *In accordance with WHO standards, we have explicitly defined severe pneumonia (including the presence of danger signs and oxygen saturation below 90%) in the revised manuscript (Lines 60–63).*
- *We have clarified that this cost-effectiveness analysis was conducted in 2024 in the revised manuscript. The study period for the underlying clinical trial was clarified in the revised manuscript (Lines 114–115).*
- *All input parameters are now cited in the final columns of Table 1 (effectiveness data) and Table 2 (cost data).*
- *Now we have explicitly stated that a decision tree model was utilized for this cost-effectiveness analysis (Line 117).*
- *To enhance methodical clarity this study was conducted and reported in accordance with the Consolidated Health Economic Evaluation Reporting Standards (CHEERS 2022) statement (*[*https://pubmed.ncbi.nlm.nih.gov/35031096/*](https://pubmed.ncbi.nlm.nih.gov/35031096/)*). This was cited in the manuscript and the cheek list was filled and upload in the supplementary file.*

*To justify the comparability of the two interventions (bCPAP vs. Low-flow oxygen), we have clarified the core assumptions of our model:*

- *We assumed that both treatment cohorts received identical care, including antibiotic regimens, feeding protocols, and routine vital sign monitoring, consistent with the Ethiopian National Severe Pneumonia Treatment Guidelines for general hospitals. What varies between group was the method of oxygen delivery ( stated Line 149 of revised manuscript).*
- *The Cost effectiveness analysis adopts a short-term horizon, tracking outcomes from hospital admission to either discharge or death.* *Long-term costs and benefits were excluded from this analysis. This rationale and its implications are now clearly stated in the methodology (Line 119) and addressed as a study limitation (Lines 399–403)*

2. The manuscript requires **greater transparency in the presentation of data, tables, and model assumptions**. The sources used in Table 1 should be clarified by distinguishing which parameters were derived from randomized controlled trials and which were based on assumptions. It would also be highly valuable to include a **supplementary file** presenting oxygen calculations, model equations, and other technical details necessary to improve understanding and reproducibility of the analysis. Table numbering should be corrected, and the base-case results should be organized into a dedicated table.

***Response***

- *We have updated Table 1 to explicitly distinguish the origin of each parameter. Specifically, we have clarified that the primary effectiveness data were derived from Randomized Controlled Trials (RCTs). To ensure full transparency, each parameter is now mapped to its specific source in the "Reference" column.*
- *Supplementary File has been provided alongside the revised manuscript. This file includes the technical details necessary for reproducibility, such as:*
- *Specific oxygen consumption calculations for both group.*
- *The underlying mathematical equations in the decision tree model.*
- *CHEERS 2022 checklist for reporting*
- *We have corrected the table numbering throughout the manuscript. The base-case results have been organized and now labeled as Table 3.*

3. The **introduction requires substantial revision**. At present, it appears weak in structure, insufficiently referenced, and lacking important contextual elements. Key concepts such as hypoxia should be clearly defined, and the magnitude and severity of the problem should be presented more robustly. The Ethiopian context should be explicitly introduced in the background section. In addition, the references should be carefully reviewed to ensure that each citation appropriately supports the claims made. The rationale for the use of bCPAP should also be better developed, including its origin, current recommendations, validation in Ethiopia, whether it has been incorporated into national clinical guidelines, and how it relates to WHO and Ethiopian Ministry of Health recommendations.

***Response***

- *Severe pneumonia and hypoxaemia are now clearly defined in the revised manuscript (Lines 60–64).*
- *The clinical importance of hypoxaemia and its association with mortality have been clarified with appropriate references.*
- *All references in the introduction have been carefully reviewed and corrected to ensure each directly supports the corresponding statement.*
- *The magnitude and mortality of pneumonia among under-five children in Ethiopia are now included (Lines 68–70), strengthening the justification for this study in the Ethiopian context.*
- *The rationale for using bCPAP has been expanded (Lines 80–98), highlighting its low cost compared to low-flow oxygen (often delivered via mechanical ventilation). We describe its origins from effective studies in various countries, including testing by our co-authors at 12 Ethiopian general hospitals from 2022–2024, which demonstrated clinical effectiveness. However, its cost-effectiveness in Ethiopia remains unknown, this gap motivated our study. this is now stated in line number 94 to 95.*
- *We also clarify that the Ethiopian MoH has not yet fully incorporated bCPAP into national paediatric clinical guidelines (line number 96.*

4. The **methods section** lacks important details regarding both the implementation of the interventions and the structure of the decision model. For example, the manuscript should clarify how and when patients were reassessed, whether blood oxygen saturation was measured, whether both groups had the same clinical severity at admission, which components were included in medical costs, whether intravenous antibiotics were administered in parallel with the interventions, why average length of hospital stay was not considered, and what happened to children initially treated as outpatients who later required admission. The absence of certain clinical branches in the decision tree, such as medical ventilation after treatment failure, should also be justified. Likewise, the manuscript should specify more clearly the type and number of hospitals included in the analysis.

***Response:***

- *In both group (bCPAP and low flow oxygen) reassessment was done at 1 hour and 4 hours after initiation, and then, for patients who respond, every 4 hours during the first 24 hours. We updated in the revised manuscript in line number 154.*
- *In both groups children who initially treated as outpatients who later required admission where excluded. And also those who have comorbidity were excluded. Now clearly stated in the revised manuscript in line number 150 to 152.*
- *In the medical cost we included in the following, Registration /consultation, Laboratory, Medicines and supplies, and Hospital bed. (Listed in table 2).*
- *The average length of hospital stay was considered in the methods section line number 212. And in Table 1 (Average Duration of intervention in bCPAP /Low-flow oxygen.*
- *Regarding the branches of decision tree, three possible braches where considered after treatment failure, death or recovery and mechanical ventilation, as per RCT protocol. The recovery branch after treatment failure was due to escalated oxygen therapy. Now stated in the note under the decision tree figure.*
- *We described the type of hospital as general (secondary) hospital. We specified this in revised manuscript in line number 113 and our conclusion also specific to general hospitals.*

5. The **results section** should be presented with greater clarity and completeness. At present, it appears too brief and somewhat imprecise. The authors are encouraged to revise the tables that contain empty cells, expand the presentation of relevant descriptive variables such as age, sex, and residence, and organize the main findings more clearly to facilitate interpretation.

***Response:***

- *We would like to clarify that the primary objective of this manuscript is to present a Cost-Effectiveness Analysis rather than the clinical outcomes of the underlying Randomized Controlled Trial (RCT). Detailed clinical findings, including relative risks and comprehensive demographic breakdowns (age, sex, treatment outcomes), have been published previously. To maintain focus on our objective, we have prioritized data essential to the cost effectiveness, costs and health impacts measured in DALYs where presented in table one as per* ***Consolidated Health Economic Evaluation Reporting Standards 2022 (CHEERS 2022) Statement*** *reporting format* [*https://pubmed.ncbi.nlm.nih.gov/35031096/*](https://pubmed.ncbi.nlm.nih.gov/35031096/)
- *The empty cells in the first row of the base-case results represent the baseline comparator (Low-flow oxygen). By definition, incremental values, such as Incremental Cost and Incremental DALYs averted, are calculated as the difference between the intervention (bCPAP) and the comparator.*

*Therefore, these cells remain empty in the first row to signify the* ***reference point. In*** *the second row (bCPAP), the values are correctly populated as follows:*

- *Inc. Cost: $125.33 - 110.83 = $14.50$*
- *Inc. DALYs averted: $0.1163 - 0.0124 = 0.1039$*

*This formatting is standard practice in economic evaluation as per the* ***CHEERS Statement*** *reporting format.*

6. The **discussion section** requires major strengthening. It should focus more directly on the study findings and avoid conflating clinical effectiveness with cost-effectiveness. A deeper and better-justified interpretation of the findings is needed, particularly regarding the apparently high cost-effectiveness of strategies such as nurse support provided by telephone. The discussion should engage more critically with the existing literature, provide a stronger explanation of the results, and offer a more convincing justification for the appropriateness and implications of the analysis.

***Response:***

- *In the revised manuscript now focused on the objective of the study.*
- *Regarding the clinical effectiveness with cost-effectiveness of strategies such as nurse support provided by telephone in Malawi. The conflict arises from two separate studies conducted at in Malawi that looks the same:*
- *The cost-effectiveness analysis study (2017) (reference 32) used effectiveness data from Bangladesh, not Malawi*
- *A later 2019 RCT in Malawi (reference 14) found bCPAP no clinical effectiveness.*

*The reviewers consider these as the same study, that reported no clinical effectiveness but found cost effective. To address this, we added the following clarification in the revised manuscript (line 356): "Note that the cost-effectiveness analysis (ref 32) drew on Bangladesh effectiveness data from 2017, while the Malawi RCT (ref 14) conducted in 2019 reported no clinical benefit."*

- *We focused on more on existing literature in LMICs and stated Implication of these studies in line 386 to 398 in the discussion section.*

7. Several **additional technical improvements** are recommended to enhance the robustness of the manuscript: updating the GDP value to 2025, expanding the probabilistic sensitivity analysis to 10,000 iterations, and considering a five-year scenario analysis to better estimate long-term DALYs, since the current approach may underestimate the value of the intervention.

***Response:***

- *The trial was conducted starting from 2022, so we used this as base year for this cost effectiveness analysis. To compare our cost effectiveness against WHO-ChOICE threshold of one time GDP per capita we have to use the same year of threshold when cost occurs which is 2022.*
- *The probabilistic sensitivity analysis we updated the 10,000 iteration. And now changed in figure 3, 10,000 iteration provides good estimate of uncertainties in the results.*
- *Although we agree that* a five-year scenario analysis to better estimate long-term cost and effect, *we did not perform a five-year scenario analysis because the study utilized a decision tree model, which is inherently designed for shorter time horizons rather than longitudinal flow. Consequently, long-term effects of the intervention were not captured. Furthermore, as the original trial did not follow patients beyond the recovery point or doesn’t have five-year follow-up, these long-term outcomes remain outside our current scope. We have explicitly detailed these constraints in the limitations in the discussion section.*

**Point by point response to Reviewer #1:** 

1. Define severe pneumonia using WHO criteria (danger signs + SpO2<90%)

***Response:*** *Severe pneumonia using WHO criteria was defined in the revised manuscript in line number 60 to 63.*

2. Add 5-year scenario analysis for long-term DALYs (currently underestimates value)

- ***Response:*** *Although we agree that a five-year scenario analysis to better estimate long-term cost and effect, we did not perform a five-year scenario analysis because the study utilized a decision tree model, which is inherently designed for shorter time horizons rather than longitudinal flow. Consequently, long-term effects of the intervention were not captured. Furthermore, as the original trial did not follow patients beyond the recovery point or doesn’t have five-year follow-up, these long-term outcomes remain outside our current scope. We have explicitly detailed these constraints in the limitations in the discussion section.*

3. Clarify Table 1 sources (RCT vs. assumptions) + create Supplementary File 1 (oxygen calculations, model equations)

***Response:*** *Data related to table 1 on the effectiveness of intervention of from RCT conducted in Ethiopia and similar context. We didn’t use assumption in the table 1. Now the revised manuscript it is corrected.*

4. Update GDP to 2025 (~$1,120) + extend PSA to 10k iterations

***Response:*** *The trial was conducted starting from 2022, so we used this as base year for this cost effectiveness study. To compare our cost effectiveness against WHO-ChOICE threshold of one times GDP per capita we have to use the same base year of threshold when cost occurs which is 2022.*

*The probabilistic sensitivity analysis we updated the 10,000 iteration. And now changed in figure. 10,000 iteration provides good estimate of uncertainties in the results.*

5. Fix Table numbering (base-case results need dedicated Table 3)

***Response:*** *Now table numbering problem is fixed in all table*

6. Methods conceptually strong minor transparency fixes will make this publication ready.

***Response:*** *To improve its transparency we added the Consolidated Health Economic Evaluation Reporting Standards 2022 (CHEERS 2022) Statement reporting format, give reference for all input parameter in table 1 and 2. Also supplementary file is uploaded*

**Point by point response to Reviewer #2:**

1. Why the second author become the corresponding author and If so why he is not ordered first?

***Response:*** *The corresponding author is now a primary authors. It was previously in a order of contribution, now updated depending on contribution.*

2. The abstract is good. But the background lack place E.g. Ethiopia. The methods have no study period and avoid bracketing. It also fails to mention the models used to evaluate it why? And lacks completeness. Revisit it. The result is good but needs further refinement and the conclusion is short.

***Response:***

- *We included the study place (Ethiopia) in line number 30 and 31 in abstract section of revised manuscript.*
- *The cost effectiveness was conducted after the trial ended in December 2024, stated in the revised manuscript line number 114. But we used the cost base year of 2022 for RCT. In the abstract we also added this as “The study was conducted in 2024, cost data was adjusted to 2022 US$ (base year of the study).” Since this study is a decision analytical model it use data from different sources in different time. Most of the cost occurred in 2022.*
- *We now mentioned the Model “decision tree” in the abstract section*
- *We revised the conclusion and added the recommendation sentence related to the study conclusion*

3. Introduction is very weak and weakly referenced. First, it Fails to define hypoxia. Second, weaklybotlrganized and hade missing link. Third, the magnitude and the severity is weakly stated. Forth, did refrence number three works for the three that is the South Asia, Subject saharan Africa and Ethiopia meaning Gebre is an Asian and African and Ethiopian and have defined it?
   -Likewise, when was both interventions begin, is that similar, for how long does they work, bCPAP recommendation, what is wrong with the WHo recommendation?, Who recommended bCPAP, when, why, how, by whom, for whom, in what settings, is that validated in Ethiopia and putted in the clinical guideline? What are the recent recommendations busy wHO and Ministry health of Ethiopia and what remains unanswered and is this the best way to estimate the cost effectiveness analysis and why?

***Response***

*•	Severe pneumonia and hypoxaemia are now clearly defined (Lines 60–64).*

*•	The clinical importance of hypoxaemia and its association with mortality have been clarified with appropriate references.*

*•	All references in the introduction have been carefully reviewed and corrected to ensure each directly supports the corresponding statement.*

*•	The magnitude and mortality of pneumonia among under-five children in Ethiopia are now included (Lines 68–70), strengthening the justification for this study in the Ethiopian context.*

*•	The rationale for using bCPAP has been expanded (Lines 81–98), highlighting its low cost compared to low-flow oxygen (often delivered via mechanical ventilation). We describe its origins from effective studies in various countries, including RCT by our co-authors at 12 Ethiopian general hospitals from 2022–2024, which demonstrated clinical effectiveness. However, its cost-effectiveness in Ethiopia remains unknown, this gap motivated our study. We also clarify that the Ethiopian Ministry of Health (MoH) has not yet fully incorporated bCPAP into national paediatric clinical guidelines, despite its proven clinical efficacy locally*

4. Methods. It is relatively good but it is incomplete for some issues.
   -The study period is missed. Likewise, you stated that you have used published literatures. Are they up to date?.

***Response:*** *Study period is 2024, now stated in the line number 114 to 115 similarly the time when the trial run was also stated.*

-bCPAP is incompletely presented E.g when was the reassessment done for the admitted underfive child?

***Response:*** *In the revised manuscript we clearly stated when the reassessment was done for both group it was similar now corrected in line number 152 to 154.*

--Are both procedures comparable?

***Response:*** *both group were comparable in antibiotic treatment, feeding, and routine assessment of vital signs as directed by the Ethiopian National Severe Pneumonia Treatment Guidelines at the general hospital level. What vary was the only in oxygen delivery methods by bCPAP or low flow oxygen as per trial protocol. This was stated in revised manuscript line number 148 to 150..*

-How was confounding factors in the trial? What type of specific RCT?

***Response:*** *The trial was a pragmatic cluster-randomised controlled. Potential confounders like age was adjusted in the trial. In the revised manuscript stated in line number 110.*

-On the decision tree model, why there is no direct medical ventilation after treatment failure?

***Response:*** *Regarding the branches of decision tree, three possible braches where considered after treatment failure, death or recovery and mechanical ventilation, as per RCT protocol. The recovery branch after treatment failure was due to escalated oxygen therapy. Now stated in the note under the decision tree figure.*

-The components of medical costs are not described?

***Response:*** *Now in the revised manuscript in the table 2 components of medical cost like described are Registration/ consultation Laboratory, Medicines and supplies and Hospital bed were listed*

-Is the child at the same severity of (severe pneumonia) illness at admission for both interventions?

***Response:*** *Yes, all patient first have to be diagnosed with severe pneumonia as shown in the start of decision tree model it consider children with severe pneumonia.*
-Is there blood oxygen measurement in the assessment?

***Response:*** *yes during reassessment it was assessed by pulse oximetry*
-What are those general hospitals and their numbers and are they not on trnsition to Specialized or comperherensive hospitals?

***Response:*** *The selected hospitals were equipped with mechanical ventilation capabilities. This study did not consider patient transfers to other facilities (e.g., specialized or comprehensive hospitals) or their subsequent outcomes. Such transfers were treated as treatment failures at the general hospitals*.

-Is the diagnosis at OPD or inpatient and who diagnosed the under five children? This is also true for the discharge diagnosis?

***Response:*** *In the trial we considered only severe pneumonia these are treated inpatient.*

-Is that parallel treatment with the IV antibiotics and the interventions or not?

***Response:*** *Yes there is parallel treatment like IV antibiotics , feeding, and routine assessment of vital signs. These were similar for both bCPAP and low oxygen flow group.* *We stated this in the manuscript line number 148 to 150*.

-Why didn't you consider the mean hospital stay for both?

***Response:*** *We considered this in line number 212 of revised manuscript*
-How many of these underfive children were treated at OPD and failedand then admitted?

***Response:*** *All included patients had severe pneumonia and received treatment upon admission, they were not previously treated at the outpatient department (OPD). Patients who received any treatment at OPD and subsequently failed were excluded from this study.*

-What are the assumptions of cost effectiveness analysis? And are they complete?

***Response****: The key assumptions of our cost-effectiveness analysis are now clearly stated (Lines 121, 149, 397–398):*

- *Both treatment cohorts (bCPAP and low-flow oxygen) received identical care except for the oxygen delivery method, including the same antibiotic regimens, feeding protocols, and routine vital sign monitoring per Ethiopian National Severe Pneumonia Treatment Guidelines.*
- *The analysis uses a short-term horizon (hospital admission to discharge or death); long-term costs and benefits were excluded.*

-How quality was the data?

***Response:*** *Since most of input parameter we used for this study is from RCT so it has a good quality.* *. To address parameter uncertainty, we conducted sensitivity analyses, which consistently showed bCPAP remaining cost-effective across plausible ranges.*

-What is rhe assumptions behind the analysis and convince your reader for the appropriateness of the analysis?
-Some lacks reference.

***Response****:*

*This paper follows the standard reporting format of* ***Consolidated Health Economic Evaluation Reporting Standards 2022 (CHEERS 2022) Statement.*** The design follow the format. We referenced at and supplied this as supplementary file.

*The primary aim of this study is to evaluate bCPAP's cost-effectiveness following our prior clinical effectiveness evaluation. The key assumption—that bCPAP serves as a cost-effective alternative to low-flow oxygen—is directly tested and confirmed by our findings*

*All input parameters are fully referenced in Tables 1 and 2 (reference columns) in the revised manuscript.*

5. Results
   -It is short not clear, brief and accurate.
   -Why some columns of the table are empty? E.g Table 1.
   -What about the other variables e.g Age, sex, residence?

***Response:***

*We would like to clarify that the primary objective of this manuscript is to present a Cost-Effectiveness Analysis rather than the clinical outcomes of the underlying Randomized Controlled Trial (RCT). Detailed clinical findings, including relative risks and comprehensive demographic breakdowns (age, sex, treatment outcomes), have been published previously. To maintain focus on our objective, we have prioritized data essential to the cost effectiveness, costs and health impacts measured in DALYs where presented in table one as per* ***Consolidated Health Economic Evaluation Reporting Standards 2022 (CHEERS 2022) Statement*** [*https://pubmed.ncbi.nlm.nih.gov/35031096/*](https://pubmed.ncbi.nlm.nih.gov/35031096/)

*The empty cells in the first row of the base-case results represent the baseline comparator (Low-flow oxygen). By definition, incremental values, such as Incremental Cost and Incremental DALYs averted, are calculated as the difference between the intervention (bCPAP) and the comparator.*

*Therefore, these cells remain empty in the first row to signify the reference point. In the second row (bCPAP), the values are correctly populated as follows:*

- *Inc. Cost: $125.33 - 110.83 = $14.50$*
- *Inc. DALYs averted: $0.1163 - 0.0124 = 0.1039$*

*This formatting is standard practice in economic evaluation as per the* ***Consolidated Health Economic Evaluation Reporting Standards 2022 (CHEERS 2022) Statement*** *reporting format*

6. Discussion
   -It is Inadequate and fails to address the objective of the study well. In addition, the clinical effectiveness is different from cost effectiveness, therefore, focus on your findings. Likewise, why advice over phone to the nurse resulted in to high cost effectiveness needs fetail explanation. Again, itbis inadequately written, short, weakly explained and justified as well as referenced. Focus on the objective of the study. I think the researchers have no full knowledge about both the interventions and failed to read the guideline.
   Regards

***Response:***

- *We revised our discussion section and stated our objective in the start of the discussion section. We stated why robust economic evidence is now essential to guide national adoption and policy decisions. In the first section of paragraph. In the revised manuscript now focused on the objective of the study.*
- *Regarding the clinical effectiveness and cost-effectiveness of strategies such as nurse support provided by telephone in Malawi. The conflict arises from two separate studies conducted at in Malawi that looks the same:*
- *The cost-effectiveness analysis study (2017) (reference 32) used effectiveness data from Bangladesh, not Malawi*
- *A later 2019 RCT in Malawi (reference 14) found that bCPAP have no clinical effectiveness.*
- *The reviewers consider these as the same study, that reported no clinical effectiveness but found cost effective. To address this, we added the following clarification (possible explanation) in the revised manuscript (line 356): "Note that the cost-effectiveness analysis (ref 32) drew on Bangladesh effectiveness data from 2017, while the Malawi RCT (ref 14) conducted in 2019 reported no clinical benefit."*
- *We focused on more on existing literature in LMICs and stated Implication of these studies in line 386 to 398 in the discussion section of the revised manuscript.*
