# Supplementary material for: Cost-effectiveness of bubble continuous positive airway pressure in treating severe pneumonia and hypoxaemia in under-five children in Ethiopia
Source: PLoS One. 2026 Jun 23;21(6):e0352122. doi: 10.1371/journal.pone.0352122 (PMC13289904; doi:10.1371/journal.pone.0352122)
Supplement: Attachment — Submitted filename: PLOS_ONE_Peer_Review_Comments_PONE-D-26-01167R1_clean.docx [file pone.0352122.s005.docx]

**Peer Review Comments for Authors**

**Manuscript No.: PONE-D-26-01167R1**

*Title: Cost-effectiveness of bubble continuous positive airway pressure in treating severe pneumonia and hypoxaemia in under-five children in Ethiopia*

# Overall assessment

This manuscript addresses an important public health and health economic question in a resource-limited setting. The study evaluates the cost-effectiveness of locally made bubble continuous positive airway pressure (bCPAP) compared with WHO-recommended low-flow oxygen therapy for under-five children with severe pneumonia and hypoxaemia in Ethiopian general hospitals.

The revised manuscript has substantially improved in conceptual clarity, model description, intervention comparability, cost-input transparency, and discussion of assumptions and limitations. The use of a decision-tree model, clearer definitions of severe pneumonia and hypoxaemia, reference to CHEERS 2022 reporting, and expanded sensitivity analysis strengthen the manuscript. The findings appear relevant for policy and implementation decisions in Ethiopia and similar low-resource settings.

The conclusions are generally supported by the presented results, particularly the reported ICER of approximately US$139.5 per DALY averted and the finding that bCPAP remains cost-effective under the stated willingness-to-pay thresholds.

# Comments for the authors

- **Scientific contribution:** The topic is timely and relevant. The manuscript provides useful economic evidence to complement the existing clinical trial evidence on bCPAP in Ethiopia. This is valuable because adoption of bCPAP in national paediatric care requires not only clinical effectiveness data but also clear cost-effectiveness evidence.
- **Methods and transparency:** The methods are now clearer, particularly regarding the decision-tree structure, the short-term time horizon from admission to discharge or death, the comparability of care between groups, and the exclusion of long-term costs and outcomes. The addition of technical details and the CHEERS 2022 checklist is appropriate and improves reproducibility.
- **Interpretation:** The discussion is improved and more focused on the study objective. The manuscript appropriately acknowledges that cost-effectiveness findings should be interpreted in the context of implementation requirements, including trained staff, oxygen availability, monitoring, and facility readiness.

# Minor issues to address before acceptance

1. The manuscript should undergo careful final proofreading to correct typographical errors, grammatical issues, and awkward sentence structures. Examples include inconsistent spelling of hypoxaemia/hypoxemia and minor wording problems in the Methods and Discussion sections.
2. Please ensure consistency between the Methods and Results regarding the number of probabilistic sensitivity analysis iterations. The Results and figures report 10,000 iterations, while the Methods section should also consistently state 10,000 iterations if that is the final analysis.
3. The rationale for using 2022 as the cost year and GDP reference year is acceptable, but it should be stated consistently in the abstract, methods, results, and discussion to avoid confusion.
4. Tables and figures should be reviewed for formatting consistency, including clear labels, abbreviations, and spacing. Any empty cells should be clearly marked as not applicable, baseline comparator, or reference category where appropriate.
5. The conclusion should remain appropriately cautious and limited to Ethiopian general hospital settings. Any recommendation for scale-up should be linked to adequate training, supervision, oxygen supply, and implementation monitoring.

# Recommendation

**Minor revision.** The manuscript is scientifically relevant and methodologically acceptable for PLOS ONE after final proofreading, consistency checks, and removal of formatting artifacts. The remaining issues are mainly editorial and consistency-related rather than fundamental methodological concerns
