# Supplementary material for: Cost-effectiveness of bubble continuous positive airway pressure in treating severe pneumonia and hypoxaemia in under-five children in Ethiopia
Source: PLoS One. 2026 Jun 23;21(6):e0352122. doi: 10.1371/journal.pone.0352122 (PMC13289904; doi:10.1371/journal.pone.0352122)
Supplement: Attachment — Submitted filename: Point by point response.docx [file pone.0352122.s007.docx]

# Point by point response

**Dear Editor and Reviewers,**

Thank you very much for your time, encouraging feedback, and constructive comments on our revised manuscript. We are pleased to note your acknowledgment that the paper has substantially improved in conceptual clarity, cost transparency, and methodological rigor, and that its findings are highly relevant for health policy and implementation decisions in low-resource settings.

We have carefully addressed all the remaining minor editorial and consistency issues you raised. Below is our point-by-point response detailing the specific modifications made to the manuscript.

1. The manuscript should undergo careful final proofreading to correct typographical errors, grammatical issues, and awkward sentence structures. Examples include inconsistent spelling of hypoxaemia/hypoxemia and minor wording problems in the Methods and Discussion sections.

***Response:*** *We have thoroughly proofread the manuscript to correct typographical errors, grammatical issues, and awkward phrasing. Specifically, we standardized the spelling to "hypoxaemia" throughout the entire text. Some grammatical corrections include the following:*

- *Changed "children under five of age" to "children under five years of age" (Paragraph 1).*
- *Changed "one times the per capita GDP" to "one times the GDP per capita".*
- *Revised "Receiving bCPAP comparing low flow oxygen therapy" to "receiving bCPAP compared with low-flow oxygen therapy" (Paragraph 6).*
- *Revised "Concluded the context-dependent nature" to "concluded that the efficacy of bCPAP is context-dependent" (Paragraph 7).*
- *Corrected "Context-specific implementation studies... ascertain the potential..." to "Context-specific implementation studies... are needed to ascertain the potential..."*

2. Please ensure consistency between the Methods and Results regarding the number of probabilistic sensitivity analysis iterations. The Results and figures report 10,000 iterations, while the Methods section should also consistently state 10,000 iterations if that is the final analysis.

***Response:*** *We have ensured consistency across the manuscript; both the Methods and Results sections now uniformly report 10,000 iterations for the probabilistic sensitivity analysis.*

3. The rationale for using 2022 as the cost year and GDP reference year is acceptable, but it should be stated consistently in the abstract, methods, results, and discussion to avoid confusion.

***Response:*** *To avoid any confusion, we have cross-checked and standardized the reference year. The cost year and GDP reference year are now consistently explicitly stated as 2022 across the Abstract, Methods, Results, and Discussion sections.*

4. Tables and figures should be reviewed for formatting consistency, including clear labels, abbreviations, and spacing. Any empty cells should be clearly marked as not applicable, baseline comparator, or reference category where appropriate.

***Response:*** *Tables and figures have been carefully reviewed for formatting, labeling, and consistency. All previously empty cells have been explicitly labeled as "Ref." to clearly indicate the reference intervention/comparator.*

5. The conclusion should remain appropriately cautious and limited to Ethiopian general hospital settings. Any recommendation for scale-up should be linked to adequate training, supervision, oxygen supply, and implementation monitoring.

***Response:*** *In line with the reviewer's recommendation, we have revised the conclusion to remain appropriately cautious and specific to Ethiopian general hospital settings. recommendations for scaling up the intervention have been explicitly linked to the availability of adequate training, supervision, a reliable oxygen supply, and robust implementation monitoring. These changes are reflected in the final paragraph of the Conclusion section.*
