# Supplementary material for: Cost-effectiveness of bubble continuous positive airway pressure in treating severe pneumonia and hypoxaemia in under-five children in Ethiopia
Source: PLoS One. 2026 Jun 23;21(6):e0352122. doi: 10.1371/journal.pone.0352122 (PMC13289904; doi:10.1371/journal.pone.0352122)
Supplement: Peer review history

# Peer review: Decision Letter 0

**DOI:** 10.1371/journal.pone.0352122.r001

**Authors:** Oriana Rivera-Lozada de Bonilla

12 Mar 2026

PONE-D-26-01167

Cost-effectiveness of bubble continuous positive airway pressure in treating severe pneumonia and hypoxaemia in under-five children in Ethiopia

PLOS One

Dear Dr. Negasa,

Thank you for submitting your manuscript to PLOS ONE. After careful consideration, we feel that it has merit but does not fully meet PLOS ONE’s publication criteria as it currently stands. Therefore, we invite you to submit a revised version of the manuscript that addresses the points raised during the review process.

The manuscript addresses a topic of high clinical and public health relevance, as it evaluates the cost-effectiveness of two procedures for the management of severe pneumonia in children under five years of age, with potential implications for child survival and decision-making in settings such as Ethiopia. Overall, the study has a valuable conceptual foundation and addresses an important issue; however, it requires strengthening in several methodological, reporting, transparency, and scientific argumentation aspects before it can be considered ready for publication.

First, the study would benefit from **greater conceptual and methodological clarity** . It is essential to define **severe pneumonia**  precisely according to recognized criteria, ideally based on WHO standards, including danger signs and oxygen saturation below 90%. The **study period** , the type and recency of the published sources used, and the analytical model applied should also be described more clearly. In addition, the manuscript should provide a stronger justification for the comparability of the two interventions, better describe the clinical trial or source evidence used, and explain more explicitly the assumptions underlying the cost-effectiveness analysis.

Second, the manuscript requires **greater transparency in the presentation of data, tables, and model assumptions** . The sources used in Table 1 should be clarified by distinguishing which parameters were derived from randomized controlled trials and which were based on assumptions. It would also be highly valuable to include a **supplementary file**  presenting oxygen calculations, model equations, and other technical details necessary to improve understanding and reproducibility of the analysis. Table numbering should be corrected, and the base-case results should be organized into a dedicated table.

Third, the **introduction requires substantial revision** . At present, it appears weak in structure, insufficiently referenced, and lacking important contextual elements. Key concepts such as hypoxia should be clearly defined, and the magnitude and severity of the problem should be presented more robustly. The Ethiopian context should be explicitly introduced in the background section. In addition, the references should be carefully reviewed to ensure that each citation appropriately supports the claims made. The rationale for the use of bCPAP should also be better developed, including its origin, current recommendations, validation in Ethiopia, whether it has been incorporated into national clinical guidelines, and how it relates to WHO and Ethiopian Ministry of Health recommendations.

Fourth, the **methods section**  lacks important details regarding both the implementation of the interventions and the structure of the decision model. For example, the manuscript should clarify how and when patients were reassessed, whether blood oxygen saturation was measured, whether both groups had the same clinical severity at admission, which components were included in medical costs, whether intravenous antibiotics were administered in parallel with the interventions, why average length of hospital stay was not considered, and what happened to children initially treated as outpatients who later required admission. The absence of certain clinical branches in the decision tree, such as medical ventilation after treatment failure, should also be justified. Likewise, the manuscript should specify more clearly the type and number of hospitals included in the analysis.

Fifth, the **results section**  should be presented with greater clarity and completeness. At present, it appears too brief and somewhat imprecise. The authors are encouraged to revise the tables that contain empty cells, expand the presentation of relevant descriptive variables such as age, sex, and residence, and organize the main findings more clearly to facilitate interpretation.

Sixth, the **discussion section**  requires major strengthening. It should focus more directly on the study findings and avoid conflating clinical effectiveness with cost-effectiveness. A deeper and better-justified interpretation of the findings is needed, particularly regarding the apparently high cost-effectiveness of strategies such as nurse support provided by telephone. The discussion should engage more critically with the existing literature, provide a stronger explanation of the results, and offer a more convincing justification for the appropriateness and implications of the analysis.

Finally, several **additional technical improvements**  are recommended to enhance the robustness of the manuscript: updating the GDP value to 2025, expanding the probabilistic sensitivity analysis to 10,000 iterations, and considering a five-year scenario analysis to better estimate long-term DALYs, since the current approach may underestimate the value of the intervention.

In summary, the manuscript has merit, relevance, and publication potential; however, it requires substantial revision to improve its methodological consistency, analytical transparency, argumentative depth, and overall scientific writing quality. With these revisions, the study could be significantly strengthened and brought closer to a publishable standard.

Please submit your revised manuscript by Apr 26 2026 11:59PM. If you will need more time than this to complete your revisions, please reply to this message or contact the journal office at plosone@plos.org. When you're ready to submit your revision, log on to https://www.editorialmanager.com/pone/ and select the 'Submissions Needing Revision' folder to locate your manuscript file.

Please include the following items when submitting your revised manuscript:

- A letter that responds to each point raised by the academic editor and reviewer(s). You should upload this letter as a separate file labeled 'Response to Reviewers'.
- A marked-up copy of your manuscript that highlights changes made to the original version. You should upload this as a separate file labeled 'Revised Manuscript with Track Changes'.
- An unmarked version of your revised paper without tracked changes. You should upload this as a separate file labeled 'Manuscript'.

If you would like to make changes to your financial disclosure, please include your updated statement in your cover letter. Guidelines for resubmitting your figure files are available below the reviewer comments at the end of this letter.

If applicable, we recommend that you deposit your laboratory protocols in protocols.io to enhance the reproducibility of your results. Protocols.io assigns your protocol its own identifier (DOI) so that it can be cited independently in the future. For instructions see: https://journals.plos.org/plosone/s/submission-guidelines#loc-laboratory-protocols. Additionally, PLOS ONE offers an option for publishing peer-reviewed Lab Protocol articles, which describe protocols hosted on protocols.io. Read more information on sharing protocols at https://plos.org/protocols?utm_medium=editorial-email&utm_source=authorletters&utm_campaign=protocols.

We look forward to receiving your revised manuscript.

Kind regards,

Oriana Rivera-Lozada de Bonilla

Academic Editor

PLOS One

Journal Requirements:

When submitting your revision, we need you to address these additional requirements.

1. Please ensure that your manuscript meets PLOS ONE's style requirements, including those for file naming. The PLOS ONE style templates can be found at

https://journals.plos.org/plosone/s/file?id=wjVg/PLOSOne_formatting_sample_main_body.pdf and https://journals.plos.org/plosone/s/file?id=ba62/PLOSOne_formatting_sample_title_authors_affiliations.pdf

2. Please provide additional details regarding participant consent. In the ethics statement in the Methods and online submission information, please ensure that you have specified (1) whether consent was informed and (2) what type you obtained (for instance, written or verbal, and if verbal, how it was documented and witnessed). If your study included minors, state whether you obtained consent from parents or guardians. If the need for consent was waived by the ethics committee, please include this information.

If you are reporting a retrospective study of medical records or archived samples, please ensure that you have discussed whether all data were fully anonymized before you accessed them and/or whether the IRB or ethics committee waived the requirement for informed consent. If patients provided informed written consent to have data from their medical records used in research, please include this information.

3. PLOS requires an ORCID iD for the corresponding author in Editorial Manager on papers submitted after December 6th, 2016. Please ensure that you have an ORCID iD and that it is validated in Editorial Manager. To do this, go to ‘Update my Information’ (in the upper left-hand corner of the main menu), and click on the Fetch/Validate link next to the ORCID field. This will take you to the ORCID site and allow you to create a new iD or authenticate a pre-existing iD in Editorial Manager.

4. Thank you for stating the following in the Competing Interests section:

[The authors have declared that no competing interests exist.].

We note that one or more of the authors are employed by a commercial company: Fenot Associates.

1. Please provide an amended Funding Statement declaring this commercial affiliation, as well as a statement regarding the Role of Funders in your study. If the funding organization did not play a role in the study design, data collection and analysis, decision to publish, or preparation of the manuscript and only provided financial support in the form of authors' salaries and/or research materials, please review your statements relating to the author contributions, and ensure you have specifically and accurately indicated the role(s) that these authors had in your study. You can update author roles in the Author Contributions section of the online submission form.

Please also include the following statement within your amended Funding Statement.

“The funder provided support in the form of salaries for authors [insert relevant initials], but did not have any additional role in the study design, data collection and analysis, decision to publish, or preparation of the manuscript. The specific roles of these authors are articulated in the ‘author contributions’ section.”

If your commercial affiliation did play a role in your study, please state and explain this role within your updated Funding Statement.

2. Please also provide an updated Competing Interests Statement declaring this commercial affiliation along with any other relevant declarations relating to employment, consultancy, patents, products in development, or marketed products, etc.

Within your Competing Interests Statement, please confirm that this commercial affiliation does not alter your adherence to all PLOS ONE policies on sharing data and materials by including the following statement: "This does not alter our adherence to PLOS ONE policies on sharing data and materials.” (as detailed online in our guide for authors http://journals.plos.org/plosone/s/competing-interests) . If this adherence statement is not accurate and there are restrictions on sharing of data and/or materials, please state these. Please note that we cannot proceed with consideration of your article until this information has been declared.

Please include both an updated Funding Statement and Competing Interests Statement in your cover letter. We will change the online submission form on your behalf.

If the reviewer comments include a recommendation to cite specific previously published works, please review and evaluate these publications to determine whether they are relevant and should be cited. There is no requirement to cite these works unless the editor has indicated otherwise.

[Note: HTML markup is below. Please do not edit.]

Reviewer's Responses to Questions

**Comments to the Author**

1. Is the manuscript technically sound, and do the data support the conclusions?

The manuscript must describe a technically sound piece of scientific research with data that supports the conclusions. Experiments must have been conducted rigorously, with appropriate controls, replication, and sample sizes. The conclusions must be drawn appropriately based on the data presented.

Reviewer #1: Partly

Reviewer #2: Partly

**********

2. Has the statistical analysis been performed appropriately and rigorously?

Reviewer #1: I Don't Know

Reviewer #2: Yes

**********

3. Have the authors made all data underlying the findings in their manuscript fully available?

The PLOS Data policy requires authors to make all data underlying the findings described in their manuscript fully available without restriction, with rare exception (please refer to the Data Availability Statement in the manuscript PDF file). The data should be provided as part of the manuscript or its supporting information, or deposited to a public repository. For example, in addition to summary statistics, the data points behind means, medians and variance measures should be available. If there are restrictions on publicly sharing data—e.g. participant privacy or use of data from a third party—those must be specified.

Reviewer #1: Yes

Reviewer #2: Yes

**********

4. Is the manuscript presented in an intelligible fashion and written in standard English?

PLOS ONE does not copyedit accepted manuscripts, so the language in submitted articles must be clear, correct, and unambiguous. Any typographical or grammatical errors should be corrected at revision, so please note any specific errors here.

Reviewer #1: Yes

Reviewer #2: Yes

**********

5. Review Comments to the Author

Please use the space provided to explain your answers to the questions above. You may also include additional comments for the author, including concerns about dual publication, research ethics, or publication ethics. (Please upload your review as an attachment if it exceeds 20,000 characters)

Reviewer #1: Dear Authors,

Good study with high policy relevance for Ethiopia. To strengthen for publication:

Key improvements needed:

Define severe pneumonia using WHO criteria (danger signs + SpO2<90%)

Add 5-year scenario analysis for long-term DALYs (currently underestimates value)

Clarify Table 1 sources (RCT vs. assumptions) + create Supplementary File 1 (oxygen calculations, model equations)

Update GDP to 2025 (~$1,120) + extend PSA to 10k iterations

Fix Table numbering (base-case results need dedicated Table 3)

Methods conceptually strong minor transparency fixes will make this publication ready.

Happy to discuss further.

Best regards,

Reviewer #2: The manuscript is very good and intends to address the cost effectiveness analysis of the two procedures for the care of severe pneumonia which will have immense contribution for the clinical practiceband public health significance in improving the life of children and fostering the survival of under five children. The following are our comments.

1.Whyv the second author become the corresponding author and If so why he is not ordered first?

2.The abstract is good. But the background lack place E.g. Ethiopia. The methods have no study period and avoid bracketing. It also fails to mention the models used to evaluate it why? And lacks completeness. Revisit it. The result is good but needs further refinement and the conclusion is short.

3.Introduction is very weak and weakly referenced. First, it Fails to define hypoxia. Second, weaklybotlrganized and hade missing link.Third, the magnitude and the severity is weakly stated. Forth, did refrence number three works for the three that is the South Asia, Subject saharan Africa and Ethiopia meaning Gebre is an Asian and African and Ethiopianand have defined it?

-Likewise, when was both interventions begin, is that similar, for how long does they work, bCPAP recommendation, whatv is wrong with the WHo recommendation?, Who recommended bCPAP, when, why, how, by whom, for whom, in what settings, is that validated in ethipoa and putted in the clinical guideline? What are the recent recommendations busy wHO and Ministry healthbof Ethiopia and what remains unanswered and is this the best way to estimate the cost effectiveness analysis and why?

4.Methods. It is relatively good but it is incomplete for some issues.

-The study period is missed . Likewise, you stated that you have used published literatures. Are they up to date?

-bCPAP is incompletely presented E.g when was the reassememt done for the admitted underfive child?

--Are both procedures comparable?

-How was confounding factors in the trial? What type of specific RCT?

-On the decision tree model, why there is no direct medical ventilation after treatment failure?

-The components of medical costs are not described?

-Is the child at the same severity of (severe pneumonia) illness at admission for both interventions?

-Is there blood oxygen measurement in the assessment?

-What are those general hospitals and their numbers and are they not on trnsition to Specialized or compherensive hospitals?

-Is the diagnosis at OPD or inpatient and who diagnosed the under five children? This is also true for the discharge diagnosis?

-Is that parallel treatment with the IV antibiotics and the interventions or not?

-Why didn't you consider the mean hospital stay for both?

-How many of these underfive children were treated at OPD and failedand then admitted?

-What are the assumptions of cost effectiveness analysis? And are they complete?

-How quality was the data?

-What is rhe assumptions behind the analysis and convince your reader for the appropriateness of the analysis?

-Some lacks reference.

5.Results

-It is short not clear, brief and accurate.

-Why some columns of the table are empty? E.g Table 1.

-What about the other variables e.g Age, sex, residence?

6.Discussion

-It is Inadequate and fails to address the objective of the study well. In addition, the clinical effectiveness is different from cost effectiveness, therefore, focus on your findings. Likewise, why advice over phone to the nurse resulted in to high cost effectiveness needs fetail explanation. Again, itbis inadequately written, short, weakly explained and justified as well as referenced. Focus on the objective of the study. I think the researchers have no full knowledge about both the interventions and failed to read the guideline.

Regards,

**********

6. PLOS authors have the option to publish the peer review history of their article (what does this mean?). If published, this will include your full peer review and any attached files.

If you choose “no”, your identity will remain anonymous but your review may still be made public.

**Do you want your identity to be public for this peer review?**  For information about this choice, including consent withdrawal, please see our Privacy Policy.

Reviewer #1: **Yes:** Emna ben jemia

Reviewer #2: No

**********

[NOTE: If reviewer comments were submitted as an attachment file, they will be attached to this email and accessible via the submission site. Please log into your account, locate the manuscript record, and check for the action link "View Attachments". If this link does not appear, there are no attachment files.]

To ensure your figures meet our technical requirements, please review our figure guidelines: https://journals.plos.org/plosone/s/figures

You may also use PLOS’s free figure tool, NAAS, to help you prepare publication quality figures: https://journals.plos.org/plosone/s/figures#loc-tools-for-figure-preparation.

NAAS will assess whether your figures meet our technical requirements by comparing each figure against our figure specifications.

Attachment Submitted filename: REVEWING ATTACHEMENTS.docx

# Author comment: Author response to Decision Letter 1

**DOI:** 10.1371/journal.pone.0352122.r002

16 Apr 2026

Point by point response to academic editor and reviewers comment

We would like to express our sincere gratitude to the editor and reviewers for their time and insightful feedback on this manuscript. Their thoughtful comments and constructive suggestions have significantly improved this work. We carefully reviewed each comment and have diligently modified our manuscript accordingly. In this response, we provide detailed justifications for the changes made, demonstrating how we have addressed the reviewers' concerns and incorporated their valuable suggestions. We deeply appreciate their dedication to enhancing the quality of this research.

We provided point by point response for each reviewer following the comments/questions in italics font type.

Comments from editor

1. The study would benefit from greater conceptual and methodological clarity. It is essential to define severe pneumonia precisely according to recognized criteria, ideally based on WHO standards, including danger signs and oxygen saturation below 90%. The study period, the type and recency of the published sources used, and the analytical model applied should also be described more clearly. In addition, the manuscript should provide a stronger justification for the comparability of the two interventions, better describe the clinical trial or source evidence used, and explain more explicitly the assumptions underlying the cost-effectiveness analysis.

Response

• In accordance with WHO standards, we have explicitly defined severe pneumonia (including the presence of danger signs and oxygen saturation below 90%) in the revised manuscript (Lines 60–63).

• We have clarified that this cost-effectiveness analysis was conducted in 2024 in the revised manuscript. The study period for the underlying clinical trial was clarified in the revised manuscript (Lines 114–115).

• All input parameters are now cited in the final columns of Table 1 (effectiveness data) and Table 2 (cost data).

• Now we have explicitly stated that a decision tree model was utilized for this cost-effectiveness analysis (Line 117).

• To enhance methodical clarity this study was conducted and reported in accordance with the Consolidated Health Economic Evaluation Reporting Standards (CHEERS 2022) statement (https://pubmed.ncbi.nlm.nih.gov/35031096/). This was cited in the manuscript and the cheek list was filled and upload in the supplementary file.

To justify the comparability of the two interventions (bCPAP vs. Low-flow oxygen), we have clarified the core assumptions of our model:

• We assumed that both treatment cohorts received identical care, including antibiotic regimens, feeding protocols, and routine vital sign monitoring, consistent with the Ethiopian National Severe Pneumonia Treatment Guidelines for general hospitals. What varies between group was the method of oxygen delivery ( stated Line 149 of revised manuscript).

• The Cost effectiveness analysis adopts a short-term horizon, tracking outcomes from hospital admission to either discharge or death. Long-term costs and benefits were excluded from this analysis. This rationale and its implications are now clearly stated in the methodology (Line 119) and addressed as a study limitation (Lines 399–403)

2. The manuscript requires greater transparency in the presentation of data, tables, and model assumptions. The sources used in Table 1 should be clarified by distinguishing which parameters were derived from randomized controlled trials and which were based on assumptions. It would also be highly valuable to include a supplementary file presenting oxygen calculations, model equations, and other technical details necessary to improve understanding and reproducibility of the analysis. Table numbering should be corrected, and the base-case results should be organized into a dedicated table.

Response

• We have updated Table 1 to explicitly distinguish the origin of each parameter. Specifically, we have clarified that the primary effectiveness data were derived from Randomized Controlled Trials (RCTs). To ensure full transparency, each parameter is now mapped to its specific source in the "Reference" column.

• Supplementary File has been provided alongside the revised manuscript. This file includes the technical details necessary for reproducibility, such as:

• Specific oxygen consumption calculations for both group.

• The underlying mathematical equations in the decision tree model.

• CHEERS 2022 checklist for reporting

• We have corrected the table numbering throughout the manuscript. The base-case results have been organized and now labeled as Table 3.

3. The introduction requires substantial revision. At present, it appears weak in structure, insufficiently referenced, and lacking important contextual elements. Key concepts such as hypoxia should be clearly defined, and the magnitude and severity of the problem should be presented more robustly. The Ethiopian context should be explicitly introduced in the background section. In addition, the references should be carefully reviewed to ensure that each citation appropriately supports the claims made. The rationale for the use of bCPAP should also be better developed, including its origin, current recommendations, validation in Ethiopia, whether it has been incorporated into national clinical guidelines, and how it relates to WHO and Ethiopian Ministry of Health recommendations.

Response

• Severe pneumonia and hypoxaemia are now clearly defined in the revised manuscript (Lines 60–64).

• The clinical importance of hypoxaemia and its association with mortality have been clarified with appropriate references.

• All references in the introduction have been carefully reviewed and corrected to ensure each directly supports the corresponding statement.

• The magnitude and mortality of pneumonia among under-five children in Ethiopia are now included (Lines 68–70), strengthening the justification for this study in the Ethiopian context.

• The rationale for using bCPAP has been expanded (Lines 80–98), highlighting its low cost compared to low-flow oxygen (often delivered via mechanical ventilation). We describe its origins from effective studies in various countries, including testing by our co-authors at 12 Ethiopian general hospitals from 2022–2024, which demonstrated clinical effectiveness. However, its cost-effectiveness in Ethiopia remains unknown, this gap motivated our study. this is now stated in line number 94 to 95.

• We also clarify that the Ethiopian MoH has not yet fully incorporated bCPAP into national paediatric clinical guidelines (line number 96.

4. The methods section lacks important details regarding both the implementation of the interventions and the structure of the decision model. For example, the manuscript should clarify how and when patients were reassessed, whether blood oxygen saturation was measured, whether both groups had the same clinical severity at admission, which components were included in medical costs, whether intravenous antibiotics were administered in parallel with the interventions, why average length of hospital stay was not considered, and what happened to children initially treated as outpatients who later required admission. The absence of certain clinical branches in the decision tree, such as medical ventilation after treatment failure, should also be justified. Likewise, the manuscript should specify more clearly the type and number of hospitals included in the analysis.

Response:

• In both group (bCPAP and low flow oxygen) reassessment was done at 1 hour and 4 hours after initiation, and then, for patients who respond, every 4 hours during the first 24 hours. We updated in the revised manuscript in line number 154.

• In both groups children who initially treated as outpatients who later required admission where excluded. And also those who have comorbidity were excluded. Now clearly stated in the revised manuscript in line number 150 to 152.

• In the medical cost we included in the following, Registration /consultation, Laboratory, Medicines and supplies, and Hospital bed. (Listed in table 2).

• The average length of hospital stay was considered in the methods section line number 212. And in Table 1 (Average Duration of intervention in bCPAP /Low-flow oxygen.

• Regarding the branches of decision tree, three possible braches where considered after treatment failure, death or recovery and mechanical ventilation, as per RCT protocol. The recovery branch after treatment failure was due to escalated oxygen therapy. Now stated in the note under the decision tree figure.

• We described the type of hospital as general (secondary) hospital. We specified this in revised manuscript in line number 113 and our conclusion also specific to general hospitals.

5. The results section should be presented with greater clarity and completeness. At present, it appears too brief and somewhat imprecise. The authors are encouraged to revise the tables that contain empty cells, expand the presentation of relevant descriptive variables such as age, sex, and residence, and organize the main findings more clearly to facilitate interpretation.

Response:

• We would like to clarify that the primary objective of this manuscript is to present a Cost-Effectiveness Analysis rather than the clinical outcomes of the underlying Randomized Controlled Trial (RCT). Detailed clinical findings, including relative risks and comprehensive demographic breakdowns (age, sex, treatment outcomes), have been published previously. To maintain focus on our objective, we have prioritized data essential to the cost effectiveness, costs and health impacts measured in DALYs where presented in table one as per Consolidated Health Economic Evaluation Reporting Standards 2022 (CHEERS 2022) Statement reporting format https://pubmed.ncbi.nlm.nih.gov/35031096/

• The empty cells in the first row of the base-case results represent the baseline comparator (Low-flow oxygen). By definition, incremental values, such as Incremental Cost and Incremental DALYs averted, are calculated as the difference between the intervention (bCPAP) and the comparator.

Therefore, these cells remain empty in the first row to signify the reference point. In the second row (bCPAP), the values are correctly populated as follows:

• Inc. Cost: $125.33 - 110.83 = $14.50$

• Inc. DALYs averted: $0.1163 - 0.0124 = 0.1039$

This formatting is standard practice in economic evaluation as per the CHEERS Statement reporting format.

6. The discussion section requires major strengthening. It should focus more directly on the study findings and avoid conflating clinical effectiveness with cost-effectiveness. A deeper and better-justified interpretation of the findings is needed, particularly regarding the apparently high cost-effectiveness of strategies such as nurse support provided by telephone. The discussion should engage more critically with the existing literature, provide a stronger explanation of the results, and offer a more convincing justification for the appropriateness and implications of the analysis.

Response:

• In the revised manuscript now focused on the objective of the study.

• Regarding the clinical effectiveness with cost-effectiveness of strategies such as nurse support provided by telephone in Malawi. The conflict arises from two separate studies conducted at in Malawi that looks the same:

• The cost-effectiveness analysis study (2017) (reference 32) used effectiveness data from Bangladesh, not Malawi

• A later 2019 RCT in Malawi (reference 14) found bCPAP no clinical effectiveness.

The reviewers consider these as the same study, that reported no clinical effectiveness but found cost effective. To address this, we added the following clarification in the revised manuscript (line 356): "Note that the cost-effectiveness analysis (ref 32) drew on Bangladesh effectiveness data from 2017, while the Malawi RCT (ref 14) conducted in 2019 reported no clinical benefit."

• We focused on more on existing literature in LMICs and stated Implication of these studies in line 386 to 398 in the discussion section.

7. Several additional technical improvements are recommended to enhance the robustness of the manuscript: updating the GDP value to 2025, expanding the probabilistic sensitivity analysis to 10,000 iterations, and considering a five-year scenario analysis to better estimate long-term DALYs, since the current approach may underestimate the value of the intervention.

Response:

• The trial was conducted starting from 2022, so we used this as base year for this cost effectiveness analysis. To compare our cost effectiveness against WHO-ChOICE threshold of one time GDP per capita we have to use the same year of threshold when cost occurs which is 2022.

• The probabilistic sensitivity analysis we updated the 10,000 iteration. And now changed in figure 3, 10,000 iteration provides good estimate of uncertainties in the results.

• Although we agree that a five-year scenario analysis to better estimate long-term cost and effect, we did not perform a five-year scenario analysis because the study utilized a decision tree model, which is inherently designed for shorter time horizons rather than longitudinal flow. Consequently, long-term effects of the intervention were not captured. Furthermore, as the original trial did not follow patients beyond the recovery point or doesn’t have five-year follow-up, these long-term outcomes remain outside our current scope. We have explicitly detailed these constraints in the limitations in the discussion section.

Point by point response to Reviewer #1:

1. Define severe pneumonia using WHO criteria (danger signs + SpO2<90%)

Response: Severe pneumonia using WHO criteria was defined in the revised manuscript in line number 60 to 63.

2. Add 5-year scenario analysis for long-term DALYs (currently underestimates value)

• Response: Although we agree that a five-year scenario analysis to better estimate long-term cost and effect, we did not perform a five-year scenario analysis because the study utilized a decision tree model, which is inherently designed for shorter time horizons rather than longitudinal flow. Consequently, long-term effects of the intervention were not captured. Furthermore, as the original trial did not follow patients beyond the recovery point or doesn’t have five-year follow-up, these long-term outcomes remain outside our current scope. We have explicitly detailed these constraints in the limitations in the discussion section.

3. Clarify Table 1 sources (RCT vs. assumptions) + create Supplementary File 1 (oxygen calculations, model equations)

Response: Data related to table 1 on the effectiveness of intervention of from RCT conducted in Ethiopia and similar context. We didn’t use assumption in the table 1. Now the revised manuscript it is corrected.

4. Update GDP to 2025 (~$1,120) + extend PSA to 10k iterations

Response: The trial was conducted starting from 2022, so we used this as base year for this cost effectiveness study. To compare our cost effectiveness against WHO-ChOICE threshold of one times GDP per capita we have to use the same base year of threshold when cost occurs which is 2022.

The probabilistic sensitivity analysis we updated the 10,000 iteration. And now changed in figure. 10,000 iteration provides good estimate of uncertainties in the results.

5. Fix Table numbering (base-case results need dedicated Table 3)

Response: Now table numbering problem is fixed in all table

6. Methods conceptually strong minor transparency fixes will make this publication ready.

Response: To improve its transparency we added the Consolidated Health Economic Evaluation Reporting Standards 2022 (CHEERS 2022) Statement reporting format, give reference for all input parameter in table 1 and 2. Also supplementary file is uploaded

Point by point response to Reviewer #2:

1. Why the second author become the corresponding author and If so why he is not ordered first?

Response: The corresponding author is now a primary authors. It was previously in a order of contribution, now updated depending on contribution.

2. The abstract is good. But the background lack place E.g. Ethiopia. The methods have no study period and avoid bracketing. It also fa

Attachment Submitted filename: Point by point response to editor and reviewers.docx

# Peer review: Decision Letter 1

**DOI:** 10.1371/journal.pone.0352122.r003

**Authors:** Oriana Rivera-Lozada de Bonilla, Oriana Rivera-Lozada de Bonilla

19 May 2026

PONE-D-26-01167R1

Cost-effectiveness of bubble continuous positive airway pressure in treating severe pneumonia and hypoxaemia in under-five children in Ethiopia

PLOS One

Dear Dr. Negasa,

Thank you for submitting your manuscript to PLOS ONE. After careful consideration, we feel that it has merit but does not fully meet PLOS ONE’s publication criteria as it currently stands. Therefore, we invite you to submit a revised version of the manuscript that addresses the points raised during the review process.

==============================

The revised manuscript has substantially improved in terms of conceptual clarity, model description, comparability of the interventions, transparency of cost inputs, and discussion of assumptions and limitations. The use of a decision-tree model, clearer definitions of severe pneumonia and hypoxaemia, reference to the CHEERS 2022 reporting standards, and an expanded sensitivity analysis strengthen the manuscript.

The conclusions are adequately supported by the results presented, particularly the reported ICER of approximately US$139.5 per DALY averted and the finding that bCPAP remains cost-effective under the stated willingness-to-pay thresholds. The study provides relevant economic evidence to inform policy and implementation decisions in Ethiopia and other low-resource settings.

Before acceptance, a few minor issues should be addressed: the manuscript should undergo final proofreading for style and grammar; terminology such as hypoxaemia/hypoxemia should be used consistently; the number of probabilistic sensitivity analysis iterations should be consistent across the Methods, Results, and figures; and the rationale for using 2022 as the cost year and GDP reference year should be stated consistently throughout the manuscript.

Recommendation: minor revision. The manuscript is scientifically relevant and methodologically acceptable for PLOS ONE, provided that these remaining editorial and consistency-related issues are corrected.

==============================

Please submit your revised manuscript by Jul 03 2026 11:59PM. If you will need more time than this to complete your revisions, please reply to this message or contact the journal office at plosone@plos.org. When you're ready to submit your revision, log on to https://www.editorialmanager.com/pone/ and select the 'Submissions Needing Revision' folder to locate your manuscript file.

Please include the following items when submitting your revised manuscript:

- A letter that responds to each point raised by the academic editor and reviewer(s). You should upload this letter as a separate file labeled 'Response to Reviewers'.
- A marked-up copy of your manuscript that highlights changes made to the original version. You should upload this as a separate file labeled 'Revised Manuscript with Track Changes'.
- An unmarked version of your revised paper without tracked changes. You should upload this as a separate file labeled 'Manuscript'.

If you would like to make changes to your financial disclosure, please include your updated statement in your cover letter. Guidelines for resubmitting your figure files are available below the reviewer comments at the end of this letter.

If applicable, we recommend that you deposit your laboratory protocols in protocols.io to enhance the reproducibility of your results. Protocols.io assigns your protocol its own identifier (DOI) so that it can be cited independently in the future. For instructions see: https://journals.plos.org/plosone/s/submission-guidelines#loc-laboratory-protocols. Additionally, PLOS ONE offers an option for publishing peer-reviewed Lab Protocol articles, which describe protocols hosted on protocols.io. Read more information on sharing protocols at https://plos.org/protocols?utm_medium=editorial-email&utm_source=authorletters&utm_campaign=protocols.

As the corresponding author, your ORCID iD is verified in the submission system and will appear in the published article. PLOS supports the use of ORCID, and we encourage all coauthors to register for an ORCID iD and use it as well. Please encourage your coauthors to verify their ORCID iD within the submission system before final acceptance, as unverified ORCID iDs will not appear in the published article. *Only*  the individual author can complete the verification step; PLOS staff *cannot*  verify ORCID iDs on behalf of authors.

We look forward to receiving your revised manuscript.

Kind regards,

Oriana Rivera-Lozada de Bonilla

Academic Editor

PLOS One

**Journal Requirements:**

1. If the reviewer comments include a recommendation to cite specific previously published works, please review and evaluate these publications to determine whether they are relevant and should be cited. There is no requirement to cite these works unless the editor has indicated otherwise.

2. Please review your reference list to ensure that it is complete and correct. If you have cited papers that have been retracted, please include the rationale for doing so in the manuscript text, or remove these references and replace them with relevant current references. Any changes to the reference list should be mentioned in the rebuttal letter that accompanies your revised manuscript. If you need to cite a retracted article, indicate the article’s retracted status in the References list and also include a citation and full reference for the retraction notice.

[Note: HTML markup is below. Please do not edit.]

Reviewers' comments:

Reviewer's Responses to Questions

**Comments to the Author**

1. If the authors have adequately addressed your comments raised in a previous round of review and you feel that this manuscript is now acceptable for publication, you may indicate that here to bypass the “Comments to the Author” section, enter your conflict of interest statement in the “Confidential to Editor” section, and submit your "Accept" recommendation.

Reviewer #3: All comments have been addressed

**********

2. Is the manuscript technically sound, and do the data support the conclusions?

The manuscript must describe a technically sound piece of scientific research with data that supports the conclusions. Experiments must have been conducted rigorously, with appropriate controls, replication, and sample sizes. The conclusions must be drawn appropriately based on the data presented.

Reviewer #3: Yes

**********

3. Has the statistical analysis been performed appropriately and rigorously?

Reviewer #3: Yes

**********

4. Have the authors made all data underlying the findings in their manuscript fully available?

The PLOS Data policy requires authors to make all data underlying the findings described in their manuscript fully available without restriction, with rare exception (please refer to the Data Availability Statement in the manuscript PDF file). The data should be provided as part of the manuscript or its supporting information, or deposited to a public repository. For example, in addition to summary statistics, the data points behind means, medians and variance measures should be available. If there are restrictions on publicly sharing data—e.g. participant privacy or use of data from a third party—those must be specified.

Reviewer #3: Yes

**********

5. Is the manuscript presented in an intelligible fashion and written in standard English?

PLOS ONE does not copyedit accepted manuscripts, so the language in submitted articles must be clear, correct, and unambiguous. Any typographical or grammatical errors should be corrected at revision, so please note any specific errors here.

Reviewer #3: Yes

**********

6. Review Comments to the Author

Please use the space provided to explain your answers to the questions above. You may also include additional comments for the author, including concerns about dual publication, research ethics, or publication ethics. (Please upload your review as an attachment if it exceeds 20,000 characters)

**Reviewer #3:**  Overall assessment

This manuscript addresses an important public health and health economic question in a resource-limited setting. The study evaluates the cost-effectiveness of locally made bubble continuous positive airway pressure (bCPAP) compared with WHO-recommended low-flow oxygen therapy for under-five children with severe pneumonia and hypoxaemia in Ethiopian general hospitals.

The revised manuscript has substantially improved in conceptual clarity, model description, intervention comparability, cost-input transparency, and discussion of assumptions and limitations. The use of a decision-tree model, clearer definitions of severe pneumonia and hypoxaemia, reference to CHEERS 2022 reporting, and expanded sensitivity analysis strengthen the manuscript. The findings appear relevant for policy and implementation decisions in Ethiopia and similar low-resource settings.

The conclusions are generally supported by the presented results, particularly the reported ICER of approximately US$139.5 per DALY averted and the finding that bCPAP remains cost-effective under the stated willingness-to-pay thresholds.

Comments for the authors

• Scientific contribution: The topic is timely and relevant. The manuscript provides useful economic evidence to complement the existing clinical trial evidence on bCPAP in Ethiopia. This is valuable because adoption of bCPAP in national paediatric care requires not only clinical effectiveness data but also clear cost-effectiveness evidence.

• Methods and transparency: The methods are now clearer, particularly regarding the decision-tree structure, the short-term time horizon from admission to discharge or death, the comparability of care between groups, and the exclusion of long-term costs and outcomes. The addition of technical details and the CHEERS 2022 checklist is appropriate and improves reproducibility.

• Interpretation: The discussion is improved and more focused on the study objective. The manuscript appropriately acknowledges that cost-effectiveness findings should be interpreted in the context of implementation requirements, including trained staff, oxygen availability, monitoring, and facility readiness.

Minor issues to address before acceptance

1. The manuscript should undergo careful final proofreading to correct typographical errors, grammatical issues, and awkward sentence structures. Examples include inconsistent spelling of hypoxaemia/hypoxemia and minor wording problems in the Methods and Discussion sections.

2. Please ensure consistency between the Methods and Results regarding the number of probabilistic sensitivity analysis iterations. The Results and figures report 10,000 iterations, while the Methods section should also consistently state 10,000 iterations if that is the final analysis.

3. The rationale for using 2022 as the cost year and GDP reference year is acceptable, but it should be stated consistently in the abstract, methods, results, and discussion to avoid confusion.

Recommendation

Minor revision. The manuscript is scientifically relevant and methodologically acceptable for PLOS ONE after final proofreading, consistency checks, and removal of formatting artifacts. The remaining issues are mainly editorial and consistency-related rather than fundamental methodological concerns.

**********

7. PLOS authors have the option to publish the peer review history of their article (what does this mean?). If published, this will include your full peer review and any attached files.

If you choose “no”, your identity will remain anonymous but your review may still be made public.

**Do you want your identity to be public for this peer review?**  For information about this choice, including consent withdrawal, please see our Privacy Policy.

Reviewer #3: No

**********

[NOTE: If reviewer comments were submitted as an attachment file, they will be attached to this email and accessible via the submission site. Please log into your account, locate the manuscript record, and check for the action link "View Attachments". If this link does not appear, there are no attachment files.]

To ensure your figures meet our technical requirements, please review our figure guidelines: https://journals.plos.org/plosone/s/figures

You may also use PLOS’s free figure tool, NAAS, to help you prepare publication quality figures: https://journals.plos.org/plosone/s/figures#loc-tools-for-figure-preparation.

NAAS will assess whether your figures meet our technical requirements by comparing each figure against our figure specifications.

Attachment Submitted filename: PLOS_ONE_Peer_Review_Comments_PONE-D-26-01167R1_clean.docx

# Author comment: Author response to Decision Letter 2

**DOI:** 10.1371/journal.pone.0352122.r004

25 May 2026

Point by point response

Dear Editor,

Thank you very much for your time, encouraging feedback, and constructive comments on our revised manuscript. We are pleased to note your acknowledgment that the paper has substantially improved in conceptual clarity, cost transparency, and methodological rigor, and that its findings are highly relevant for health policy and implementation decisions in low-resource settings.

We have carefully addressed all the remaining minor editorial and consistency issues you raised. Below is our point-by-point response detailing the specific modifications made to the manuscript.

1. The manuscript should undergo careful final proofreading to correct typographical errors, grammatical issues, and awkward sentence structures. Examples include inconsistent spelling of hypoxaemia/hypoxemia and minor wording problems in the Methods and Discussion sections.

Response: We have thoroughly proofread the manuscript to correct typographical errors, grammatical issues, and awkward phrasing. Specifically, we standardized the spelling to "hypoxaemia" throughout the entire text. Some grammatical corrections include the following:

• Changed "children under five of age" to "children under five years of age" (Paragraph 1).

• Changed "one times the per capita GDP" to "one times the GDP per capita".

• Revised "Receiving bCPAP comparing low flow oxygen therapy" to "receiving bCPAP compared with low-flow oxygen therapy" (Paragraph 6).

• Revised "Concluded the context-dependent nature" to "concluded that the efficacy of bCPAP is context-dependent" (Paragraph 7).

• Corrected "Context-specific implementation studies... ascertain the potential..." to "Context-specific implementation studies... are needed to ascertain the potential..."

2. Please ensure consistency between the Methods and Results regarding the number of probabilistic sensitivity analysis iterations. The Results and figures report 10,000 iterations, while the Methods section should also consistently state 10,000 iterations if that is the final analysis.

Response: We have ensured consistency across the manuscript; both the Methods and Results sections now uniformly report 10,000 iterations for the probabilistic sensitivity analysis.

3. The rationale for using 2022 as the cost year and GDP reference year is acceptable, but it should be stated consistently in the abstract, methods, results, and discussion to avoid confusion.

Response: To avoid any confusion, we have cross-checked and standardized the reference year. The cost year and GDP reference year are now consistently explicitly stated as 2022 across the Abstract, Methods, Results, and Discussion sections.

4. Tables and figures should be reviewed for formatting consistency, including clear labels, abbreviations, and spacing. Any empty cells should be clearly marked as not applicable, baseline comparator, or reference category where appropriate.

Response: Tables and figures have been carefully reviewed for formatting, labeling, and consistency. All previously empty cells have been explicitly labeled as "Ref." to clearly indicate the reference intervention/comparator.

5. The conclusion should remain appropriately cautious and limited to Ethiopian general hospital settings. Any recommendation for scale-up should be linked to adequate training, supervision, oxygen supply, and implementation monitoring.

Response: In line with the reviewer's recommendation, we have revised the conclusion to remain appropriately cautious and specific to Ethiopian general hospital settings. recommendations for scaling up the intervention have been explicitly linked to the availability of adequate training, supervision, a reliable oxygen supply, and robust implementation monitoring. These changes are reflected in the final paragraph of the Conclusion section.

Attachment Submitted filename: Point by point response.docx

# Editor report: Decision Letter 2

**DOI:** 10.1371/journal.pone.0352122.r005

**Authors:** Oriana Rivera-Lozada de Bonilla, Oriana Rivera-Lozada de Bonilla, Oriana Rivera-Lozada de Bonilla

5 Jun 2026

Cost-effectiveness of bubble continuous positive airway pressure in treating severe pneumonia and hypoxaemia in under-five children in Ethiopia

PONE-D-26-01167R2

Dear Dr. Abdi Gari Negasa,

We’re pleased to inform you that your manuscript has been judged scientifically suitable for publication and will be formally accepted for publication once it meets all outstanding technical requirements.

Within one week, you’ll receive an e-mail detailing the required amendments. When these have been addressed, you’ll receive a formal acceptance letter and your manuscript will be scheduled for publication.

An invoice will be generated when your article is formally accepted. Please note, if your institution has a publishing partnership with PLOS and your article meets the relevant criteria, all or part of your publication costs will be covered. Please make sure your user information is up-to-date by logging into Editorial Manager at Editorial Manager® and clicking the ‘Update My Information' link at the top of the page. For questions related to billing, please contact billing support.

If your institution or institutions have a press office, please notify them about your upcoming paper to help maximize its impact. If they’ll be preparing press materials, please inform our press team as soon as possible -- no later than 48 hours after receiving the formal acceptance. Your manuscript will remain under strict press embargo until 2 pm Eastern Time on the date of publication. For more information, please contact onepress@plos.org.

Kind regards,

Oriana Rivera-Lozada de Bonilla

Academic Editor

PLOS One

# Editor report: Acceptance letter

**DOI:** 10.1371/journal.pone.0352122.r006

**Authors:** Oriana Rivera-Lozada de Bonilla, Oriana Rivera-Lozada de Bonilla, Oriana Rivera-Lozada de Bonilla

PONE-D-26-01167R2

PLOS One

Dear Dr. Negasa,

I'm pleased to inform you that your manuscript has been deemed suitable for publication in PLOS One. Congratulations! Your manuscript is now being handed over to our production team.

At this stage, our production department will prepare your paper for publication. This includes ensuring the following:

* All references, tables, and figures are properly cited

* All relevant supporting information is included in the manuscript submission,

* There are no issues that prevent the paper from being properly typeset

You will receive further instructions from the production team, including instructions on how to review your proof when it is ready. Please keep in mind that we are working through a large volume of accepted articles, so please give us a few days to review your paper and let you know the next and final steps.

Lastly, if your institution or institutions have a press office, please let them know about your upcoming paper now to help maximize its impact. If they'll be preparing press materials, please inform our press team within the next 48 hours. Your manuscript will remain under strict press embargo until 2 pm Eastern Time on the date of publication. For more information, please contact onepress@plos.org.

You will receive an invoice from PLOS for your publication fee after your manuscript has reached the completed accept phase. If you receive an email requesting payment before acceptance or for any other service, this may be a phishing scheme. Learn how to identify phishing emails and protect your accounts at https://explore.plos.org/phishing.

If we can help with anything else, please email us at customercare@plos.org.

Thank you for submitting your work to PLOS ONE and supporting open access.

Kind regards,

PLOS ONE Editorial Office Staff

on behalf of

Dr. Oriana Rivera-Lozada de Bonilla

Academic Editor

PLOS One
